# Transforming Water: Social Influence Moderates Psychological, Physiological, and Functional Response to a Placebo Product

**DOI:** 10.1371/journal.pone.0167121

**Published:** 2016-11-22

**Authors:** Alia J. Crum, Damon J. Phillips, J. Parker Goyer, Modupe Akinola, E. Tory Higgins

**Affiliations:** 1 Department of Psychology, Stanford University, Stanford, California, United States of America; 2 Department of Management, Columbia Business School, New York, New York, United States of America; 3 Department of Psychology, Columbia University, New York, New York, United States of America; Technion Israel Institute of Technology, ISRAEL

## Abstract

This paper investigates how social influence can alter physiological, psychological, and functional responses to a placebo product and how such responses influence the ultimate endorsement of the product. Participants consumed a product, “AquaCharge Energy Water,” falsely-labeled as containing 200 mg of caffeine but which was actually plain spring water, in one of three conditions: a no social influence condition, a disconfirming social influence condition, and a confirming social influence condition. Results demonstrated that the effect of the product labeling on physiological alertness (systolic blood pressure), psychological alertness (self-reported alertness), functional alertness (cognitive interference), and product endorsement was moderated by social influence: participants experienced more subjective, physiological and functional alertness and stronger product endorsement when they consumed the product in the confirming social influence condition than when they consumed the product in the disconfirming social influence condition. These results suggest that social influence can alter subjective, physiological, and functional responses to a faux product, in this case transforming the effects of plain water.

## Introduction

Although we are told, “a rose by any other name would smell as sweet,” decades of psychological research establishes that the sensory properties of an object or substance are not the sole determinant of how we experience it. This is especially true for shaping experiences of food and beverage consumption, in which case simply altering the name or label of a food can literally make the product taste sweeter. For example, people enjoy the taste of Coke more when it is consumed in a brand name cup [1], children enjoy the taste of french fries, milk, and carrots more when they believe them to be from McDonald’s [2], and beer infused with vinegar is enjoyed more if it is labeled as having a “special ingredient” than when the actual ingredient is unveiled [3].

Inspired by the neurological and physiological effects found in clinical trials and placebo research [4], a growing body of research suggests that, in addition to influencing perception and behavior, mindsets and expectations can also alter more objective outcomes such as sensory experience and physiological processing. For example, increasing the perceived cost of wine results in heightened activity in areas of the brain related to pleasure and reward when consuming the exact same wine [5]. Additionally, participants who thought they were drinking an indulgent, high-calorie milkshake showed steeper declines in ghrelin, a hunger-inducing hormone, than when they thought that the same shake was a sensible, low-calorie milkshake [6].

A review of research on placebo caffeine suggests that believing one is drinking caffeinated coffee can result in increases in subjective arousal, physiological arousal, and cognitive performance even though no caffeine has been consumed [7–11] and neuroimaging studies suggest that placebo caffeine is associated with a significant bilateral dopamine release in the thalamus, therefore indicating the placebo caffeine may share some of the same dopaminergic mechanism as does actual caffeine [12]. Interestingly however, the effects of placebo caffeine are not found consistently. Some studies have found no effect of expectancy manipulations [13–15]. And some have found a counter-effect of expectancy manipulations [10, 16]. The unpredictability and variability of placebo responses in caffeine and other domains leads to an important question: what factors strengthen or weaken the power of placebo effects and thereby influence the overall experience of a product or substance?

Several moderators of the placebo response have been identified in prior research. For example, studies have shown that the effect of placebo caffeine is weakened when there is more uncertainty about whether or not one has received caffeine, as in the case of double blind manipulations (i.e., telling participants that they may have received either a placebo or the actual caffeine) [9, 17, 18]. Other studies have shown that the effect of placebo caffeine is moderated by the expectancies individuals hold about the effects of caffeine. For example, Kirsch and Weixel [17] showed that placebo caffeine (decaffeinated coffee) improved motor performance among individuals who reported at baseline that they expected it to improve performance and impaired performance for individuals who expected it to impair performance. Similarly, Fillmore and Vogel-Sprott [19] manipulated the expected effects of caffeine and found that those told to expect impairments had worse cognitive performance whereas those who were told to expect improvements had improved performance on the task. Finally, one study has shown that the expectancies of the experimenter might influence the effects of placebo caffeine. For example, Wallach [20] found that wellbeing and systolic blood pressure (SBP) were affected by placebo caffeine to a greater degree when the experimenter believed that the placebo caffeine would produce heightened alertness than when the experimenter did not believe in placebo response. Taken together, these studies suggest that the effects of placebo caffeine are moderated by the expectations of the participant and perhaps even the expectations of the experimenter.

Social influence is another potential moderator of the placebo response. Expectations and beliefs do not exist in a vacuum and instead are dynamically informed by social forces. Social forces supply new information, confirm or correct old information, and serve as a foundational source for our mindsets, beliefs, and expectations [21–25]. Individuals often turn to others for information about what foods are good or bad, what diets work or fail, and what medications are most effective. Decades of research support the idea that social forces can change subjective emotions and preferences [24, 26–30]. And more recent research has accumulated to suggest that social information (e.g., about other’s attractiveness) can increase or decrease the neurological activity in the orbitofrontal cortex and the nucleus accumbens, suggesting that social influence can produce changes in brain areas corresponding to hedonic experience [31].

One unexamined question is: can social influence literally “get under the skin” and affect physiological experiences of a product? Literature from medical and placebo research suggests that biological effects of social influence may indeed be possible. Research on mass psychogenic illness (MPI) demonstrates that the collective occurrence of self-reported physical symptoms in the absence of an identifiable pathogen is a relatively common occurrence [32]. Studies exploring MPI empirically have shown that the belief that one has inhaled a substance described as an environmental toxin, when in fact it is just odorless ambient air, can evoke the experience of corresponding symptoms (e.g., headaches, nausea, and drowsiness). Most relevant to our question of interest is evidence that these symptoms are increased (up to an 11x increase in one study) when participant are in the presence of an ostensible participant (confederate) displaying the expected symptoms [32–35].

Placebo analgesia research has also demonstrated that pain relief can be conditioned by witnessing another person receive pain relief [27]. While these medical studies suggest that social influence may affect physiological or visceral experience, they don’t actually measure physiological responses, but rather assessed them via self-reports (e.g., of pain or nausea). Further, these studies are limited to a clinical context, but it is likely that the effects of placebos have consequences for everyday life. Thus, an important question remains unanswered: can social influence moderate the physiological effects of a product or experience as indicated by objective physiological measures such as cardiovascular response?

In summary, previous research has demonstrated that beliefs and expectations about a product or experience induced by labels and other marketing actions can affect physiological and psychological responses to that product [36]. A separate body of research suggests that social influence can affect psychological responses and possibly physiological response although no study to our knowledge has directly tested the latter. Thus, it remains to be investigated whether the physiological, psychological, and functional responses to a placebo product may be moderated by social influence.

**Fig 1:**
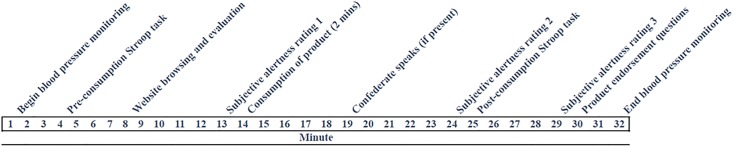
Study Timeline.

To test these questions we created a product, “AquaCharge Energy Water.” The bottle’s label indicated that the product contained 200 mg of performance and energy-enhancing caffeine. In reality, it was plain spring water. After participants consumed the product, (see Fig 1 for study timeline), we compared measures of subjective arousal, physiological arousal and cognitive function known to respond to the effects of caffeine across three conditions: a no social influence condition, in which the participant consumed the water in a room alone; a disconfirming social influence condition, in which the participant consumed the water alongside a confederate who denied the effect of the product; and a confirming social influence condition, where the confederate participant endorsed the effect of the product. In line with research on social influence, we predicted that the effects of product consumption on subjective alertness, physiological alertness, functional alertness, and product endorsement would be moderated by social influence such that the effects would be stronger with an affirming confederate and weaker in the presence of a denying confederate.

## Materials and Methods

### Participants and Design

We recruited 99 females (40% White, 36% Asian, 24% Black,) (mean age = 24 years; SD = 5) from a university study pool. The decision to have at least 30 participants in each condition was predetermined based on published research on MPI, placebo caffeine, and social influence [10, 32, 37]. Participants were screened at the onset and excluded from the study if they were a smoker, had high blood pressure, or reported intake of alcohol, antihistamines, caffeine, or anything besides water two hours prior to experiment. Participants received $20 for their participation, with the opportunity to earn a $100 gift card reward if they completed the one-week follow-up questionnaire. Participants were randomly assigned to: 1) a no social influence condition (N = 35); 2) a confirming social influence condition (N = 32); or 3) a disconfirming social influence condition (N = 32). To heighten the effect of social influence [38] and reduce variability from gender effects on arousal [39], participants were matched to the same-race and same-sex confederates. All procedures, including the consenting and debriefing process, were reviewed and approved by the University’s Institutional Review Board.

### Measures

#### Subjective Alertness

Because caffeine intake has a robust effect on subjective arousal [40, 41], subjective alertness was measured once pre-consumption (minute 13) and twice post-consumption (minutes 24 and 29) by a well-validated scale [17] for the purposes of studying the effects of caffeine (see Fig 1 for study timeline). The scale includes fifteen adjectives along a 5-point Likert scale, which factor in three subscales indicating alertness, relaxation, and tension. In the current sample, Cronbach’s alpha was adequate (alertness subscale alpha = .79; tense subscale alpha = .73; relaxation subscale alpha = .86).

#### Physiological Alertness

The most consistent effect of caffeine consumption is its effect on systolic blood pressure (SBP) [39, 42]. Therefore, we used SPB as our primary measure of physiological alertness, although we also collected diastolic blood pressure (DBP) and heart rate (HR). Participants’ systolic blood pressure was measured using a Noninvasive Blood Pressure System (NIBPH100D; Biopac Systems, Inc., Santa Barbara, CA). The NIBPH100D uses a standard upper arm cuff and a finger sensor to estimate continuous blood pressure signals. The signals were recorded with Acqknowledge software (Biopac Systems, Inc., Santa Barbara, CA). SBP responses were averaged across 1-minute epochs. A very small proportion of data (< 2%) could not be scored due to faulty sensors, loss of signal, or noisy signals; however, our models were able to account for this. To address any outliers, we used a 90% winsorization: within each minute, values at the 5^th^ percentile or below were set to the 5^th^ percentile value; values at the 95^th^ percentile or above were set to the 95^th^ percentile value. We also tested an alternative approach for dealing with outliers in which we computed the means and standard deviations separately for each of the 32 minutes, and dropped those observations that were greater than or equal to 3 standard deviations from the mean for a given minute. This approach produced consistent results with the winsorizing method (see S1 and S2 Tables).

#### Functional Alertness

Because cognitive performance can be affected by caffeine intake [43, 44], participants’ cognitive interference was measured using the Stroop color-naming task [45, 46], both pre-consumption (minutes 5–8) and post-consumption (minutes 25–28). Participants were instructed to indicate the font color of a letter string by pressing one of four appropriate color-coded keys as quickly as possible. 20 practice trials and 180 experimental trials were randomized into three variations: on incongruent trials (~60 trials) a color word appeared in a font color different from its semantic meaning (e.g., “BLUE” in red font), whereas confirming trials (~60 trials) displayed a color word that matched its font color (e.g., “BLUE” in blue font). Control trials (~60 trials) consisted of a string of “@”s in one of the four font colors. Stroop interference scores were computed as the difference in response latencies (in milliseconds) between incongruent and congruent trials, with higher scores indicating greater cognitive depletion. On the basis of procedures used in other work, incorrect responses and latencies above 2000 ms and below 200 ms were recoded as missing data [45–47].

#### Product Endorsement

To assess product endorsement, participants were asked a variety of questions pertaining to their endorsement of the AquaCharge product. First, they were asked how much they would pay for AquaCharge Energy Water in a store. To guide their answer, participants were given several benchmarks to assist them in making their decision, including Gatorade ($1.50), Poland Spring Water ($1.75), and Five Hour Energy ($2.10). Second, they were asked their likelihood of buying AquaCharge in a store (1 = not at all to 7 = extremely). Third they were asked if they would be an ambassador for the product by either working for the product or helping to make it available on or near their campus (1 = not at all to 7 = extremely). One week following their participation, participants were contacted by email and asked to fill out a short survey asking how many people they had told about the product and for each person to indicate the reasons for telling them (response options included: because a) “I love the product and want people to know about it”; b) “I am just a talker and tell everyone things”; c) “I didn’t like the product and wanted them to know I didn’t like it”; c) “I thought it was an interesting idea”; d) “I thought they would be interested in it” and; e) “other”). The number of people who were told for reason (a) or reason (d) were included as a fourth item of product endorsement. Finally, as a thank you for completing the one-week follow-up survey, participants were asked to choose whether they wanted to be entered into a lottery to win either a $100 gift card or an $80 gift card and a case of 30 bottles of AquaCharge. This behavioral preference (0 = no, 1 = yes) was included as our fifth item of product endorsement. As an overall measure of product endorsement, we created a composite index combining the items: Since each item was measured on a different metric, we first standardized them before averaging them together to form a single index. Because only 52 subjects completed the one-week follow-up survey, we created two indices of product endorsement, one that contained all five items (alpha = .66) and one that contained only the three items asked at the end of the experiment (alpha = .63). As described in more detail below, both indices produce consistent results.

#### Caffeine Exposure and Expectancies

Because caffeine exposure and caffeine expectancy have been shown to alter the effect of caffeine consumption on subjective and physiological alertness [7, 10], we measured caffeine exposure (number of days in a typical week they drink caffeine) and caffeine expectancy (11 items, e.g. “Caffeine makes me feel more energetic” (1 = very unlikely to 6 = very likely) (adapted from [10]). These measures were tested as a covariate in all analyses.

### Procedure

Participants were asked not to drink caffeine or consume anything except water for two hours prior to the onset of the one-hour study, which took place during the morning hours of 9am-12pm. The formal experiment occurred over the course of 32 carefully scripted minutes (see Fig 1 for study timeline). A female experimenter was trained to strictly follow the protocol timing and script and another experimenter, trained to operate the NIBPH100D monitor, was present during each session to help orchestrating the experiment and assure that the each measure and test was performed at precisely the right time.

**Fig 2:**
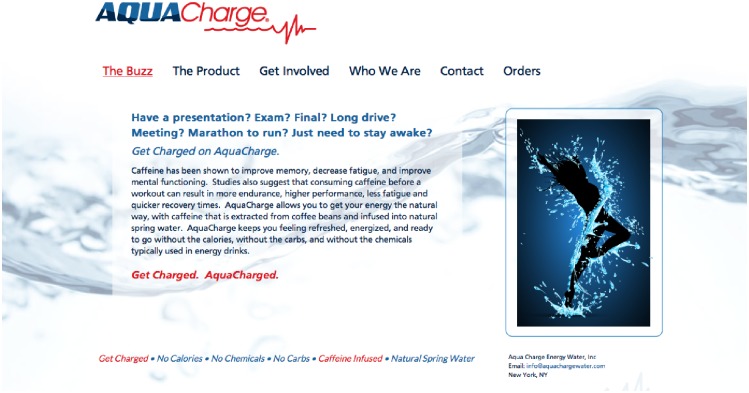
Screenshot of the AquaCharge Energy Water website “The Buzz” page. The AquaCharge Energy Water website was designed specifically for the purposes of study by Gibbs Graphics. The website for the fictional product AquaCharge Energy Water was removed from the Internet after the study was complete. This website page and other pages (S1 File) are reprinted under a CC BY license, with permission from Gibbs Graphics, original copyright 2013.

After arriving at the laboratory the experimenter checked to assure that pre-experimental instructions had been followed and reminded participants that the purpose of the study was to test a new product being developed by entrepreneurs at the University’s business school. The experimenter explained the procedures and risks documented in the consent form with the participant, following which the participant was given time to review and sign. Upon consenting, participants completed short questionnaires assessing their height, weight, caffeine exposure, and caffeine expectancies. Participants were then directed into a room in which the ostensible participant (confederate) was present (confirming and disconfirming confederate conditions) or into an empty room with no other participant present (no confederate condition). The participant and (for the two social influence conditions) the confederate were then connected to the blood pressure machine to record SBP responses after which they completed a Stroop task (minutes 4–6). Following these baseline measurements, participants were given an iPad and asked to view and rate the product’s website (minutes 8–13) (Fig 2 for “The Buzz” page; all other website pages located in S1 File). The purpose of the product ratings was to add to the credibility of the study guise (beta-testing the marketing for a new product) and ensure that the participants paid attention to the placebo/mindset intervention (that the water is infused with caffeine and thus will increase their arousal, energy and reaction time when consumed).

**Fig 3:**
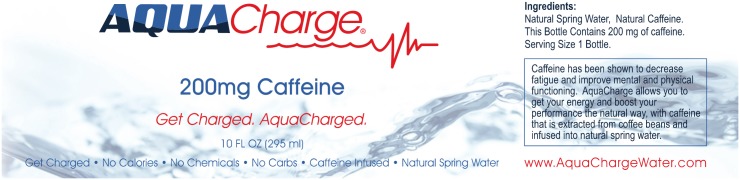
AquaCharge Energy Water label. The AquaCharge Energy Water label was designed specifically for the purposes of study by Gibbs Graphics. The label was printed and attached to 8oz plastic bottles of plain spring water containing no caffeine. This label is reprinted under a CC BY license, with permission from Gibbs Graphics, original copyright 2013.

After completing the website-rating form, participants were asked to complete the mood rating form to assess their baseline (pre-consumption) levels of subjective alertness (minute 13). At minute 14, participants were given the 8 oz bottle of AquaCharge (Fig 3) and asked to consume the product in its entirety within two minutes. Participants were then told to wait “a few minutes for the energy water to take effect.” Three minutes after consuming the water (minute 19), the confederate participant spoke. In the disconfirming confederate condition, the confederate stated: “Hmm. I don’t really feel any change. I’m definitely NOT feeling charged up. How about you?” In the confirming confederate condition, the confederate participant stated: “Wow! This is really something. This is really waking me up! How about you?” After the confederate spoke, the experimenter entered the room and reminded participants to stay quiet during the testing phase. For the next five minutes, participants sat quietly and completed a few questions asking about the product label and taste of the product (minutes 19–24). These were filler questions designed to keep them focused on the product and maintain alignment with the study guise.

Participants were then instructed to complete a subjective alertness form (minute 24), followed by the Stroop task (minutes 25–27), another subjective alertness form (minute 29), and the product endorsement survey (minutes 30–32). One week later participants were contacted by email and asked to complete a short survey asking them how many people they had told about the product and for what reason. After data was collected for all participants, subjects were debriefed via email about the true purpose of the experiment.

## Results

### Data Analytic Strategy for Physiological Measures

To assess changes in blood pressure and heart rate over time (measured once per minute from minutes 1–32), as a function of condition, we used longitudinal modeling that enabled us to account for both changes in level and changes in slope at multiple measurements, focusing on the time period beginning after they started drinking the water (minute 14) to before they took the second stroop test (minute 24). Specifically, we fit multilevel models that contained two submodels: a Level-1 submodel that described how the outcome changed over time for a given participant, and Level-2 submodels that predicted key parameters of the Level-1 model as a function of condition, controlling for blood pressure during the baseline period (minute 1 to 13). We also included time-varying predictors at Level 1 when these allowed us to test additional hypotheses beyond effects on basic linear slope; these permitted intercepts or slopes to vary by condition as a function of different time periods associated with key inflection points in the experimental procedure. Potential inflection points included minute 14, when participants first began drinking the beverage; minute 16, after they finished drinking the water; and minute 19, when the confederate first spoke in the two confederate conditions. The best fitting model contained only a shift in slope at minute 19. As a supplemental analysis, we also analyzed subjective alertness (measured at three points: minutes 13, 24, and 29) using a longitudinal multilevel model (the cross-sectional analysis for this outcome is described below). More details about model fitting are included in S1 Text.

#### Systolic Blood Pressure

For systolic blood pressure, there was a significant shift in slope at minute 19, directly after the confederate spoke, as a function of whether participants were in the disconfirming versus confirming confederate condition, *B* = -1.93, *z* = -2.79, *p* = 0.005. In the disconfirming confederate condition, the slope in SBP went from an increase, 0.69 mmHg/minute in the pre-confederate period from minutes 14–19, *B* = 0.69, *z* = 1.95, *p* = 0.051, to a significant decrease, -1.09 mmHg/minute from minutes 19–24, *B* = -1.09, *z* = -3.1, *p* = 0.002. Within the disconfirming condition this shift in slope was significant, *χ*(1) = 13.32, *p* = 0.003. However, in the confirming confederate and no confederate conditions, the slopes were not significantly different from zero in either the minutes 14–19 or the minutes 19–24 periods, *z*s<1, *p*s>0.32. Consequently, there were also no significant changes in the slope of SBP for these two conditions, *z*s<0.35, *p*s >0.70.

**Fig 4:**
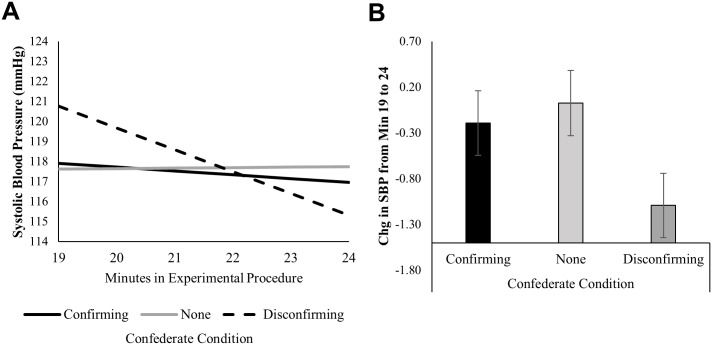
Effects of condition on changes in systolic blood pressure. Levels of systolic blood pressure in mmHg during key minutes of the experimental procedure, modeling longitudinally using multilevel linear regression. Panel **(A)** represents the longitudinal trajectory as a function of condition, focusing on the post-shared reality period, from minute 19 to minute 24, directly before the second Stroop Task. Panel **(B)** represents the magnitude of the same rate of change in blood pressure, the change in mmHg per minute, during the same period. The full longitudinal model begins at minute 14 and allows for a shift in slope as a function of condition at minute 19, when the confederate provided a verbal reaction to the product in the two social influence conditions. Level-1 predictors were initial levels of SBP at minute 14, the minute by minute rate of change from minute14-24, and a shift in the rate of change beginning at minute 19. Condition and pre-consumption levels of blood pressure (the average from minutes 1–13, grand-mean centered) were Level-2 predictors of each Level-1 parameter. For the full longitudinal models from which these means are derived, see S1 to S2 Tables. *N* = 95.

To focus more explicitly on the time period after the confederate spoke, we mean-centered time at minute 19 rather than minute 14 and ran the same longitudinal model. As predicted, there was a larger decrease in blood pressure in the disconfirming confederate condition relative to the confirming confederate condition, in the post-confederate-reaction period from minutes 19–24 (-1.09 mmHg/minute vs -0.19 mmHg/minute), a marginally significant difference, *B* = -0.90, *z* = -1.80, *p* = 0.072. When compared to the no confederate condition, only the slope in the disconfirming confederate condition significantly differed from the slope in the no confederate condition (-1.09 mmHg/minute vs 0.026 mmHg/minute), *B* = -1.12, *z* = -2.23, *p* = 0.026. The slope in the confirming confederate condition was not statistically different from the slope in the no confederate condition during this time period (-0.22 mmHg/minute vs 0.026 mmHg/minute), *B* = -0.22, *z* = -0.43, *p* = 0.666. These results indicate that the confederate’s denial of the product’s effectiveness at minute 19 was particularly impactful on blood pressure, relative to having no confederate or having a confederate endorse the product at that time. Differences in the changes in SBP from minutes 14–24 by condition are presented in Fig 4. Full regression models for this outcome are in S1 and S2 Tables. We found no effect of condition on initial levels or change over time during the period of interest (minutes 14–24) for DBP or HR.

### Data Analytic Strategy for Self-Report and Behavioral Measures

For self-report and behavioral measures, we used a single-level multiple regression model. The primary model contained two orthogonally-coded condition variables: one variable compared the average of the confederate conditions (each assigned a value of 0.5) to the no confederate condition (-1). The second variable compared the disconfirming confederate (+0.5) to the confirming confederate condition (-0.5); the no confederate condition was set to 0 for this variable. To compare each of the two social influence conditions to the no confederate condition, we also represented condition as two dummy variables, with each assigning a value of 0 to the no confederate condition (0,+1,0; 0,0,+1). S3–S7 Tables display regression coefficients for four models for each outcome, obtained using stepwise regression with the following blocks of predictors: (1) condition only, (2) addition of the baseline version of the outcome or related baseline measure, (3) addition of baseline caffeine expectancy, and (4) addition of caffeine exposure. The main text focuses on Model 2. Analyses of product endorsement controlled only for condition (Model 1), as it was not possible to measure product endorsement at baseline.

#### Subjective Alertness

**Fig 5:**
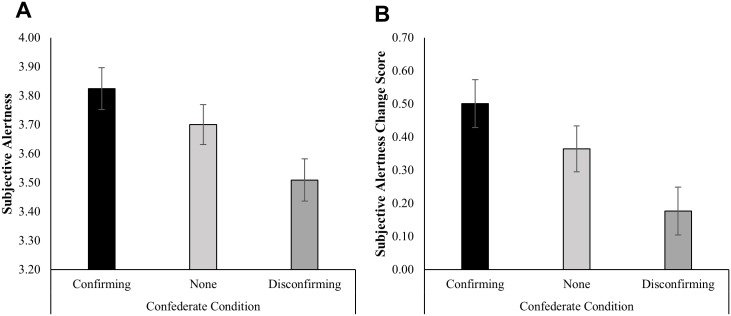
Effects of condition on subjective alertness. Error bars represent +/- 1 SE. **(A)** Average subjective alertness at min24 and min29, controlling for subjective alertness at T13, as a function of condition. **(B)** Raw change scores between post- (min24 to min29) and pre-consumption (min13) levels of subjective alertness, as a function of condition. Adjusted means were obtained from single-level linear regression. *N* = 99.

For ease of interpretation, subjective alertness at minute 24 and minute 29 were averaged together to form a single outcome measure. Controlling for baseline subjective alertness, participants in the confirming confederate condition (M_adj_ = 3.82) had higher subjective alertness than participants in the disconfirming confederate condition (M_adj_ = 3.51), *B* = 0.32, *t*(95) = -3.08, *p* = 0.003, *d* = -0.55. Subjective alertness of the no confederate condition (M_adj_ = 3.70) was in between the subjective alertness for the confirming confederate and disconfirming confederate conditions. The difference between the disconfirming confederate and no confederate was marginally significant *B* = -0.19, *t*(95) = -1.91, *p* = 0.06, *d* = -0.34, however the difference between the confirming confederate and no confederate was not statistically significant *B* = 0.12, *t*(95) = 1.24, *p* = 0.218, *d* = 0.22. Raw differences in the changes in subjective alertness from baseline to post consumption by condition are presented in Fig 5. The full regression models for this outcome are in S3 Table. Results were consistent when subjective alertness at minutes 24 and 29 were analyzed separately (S4 Table) and also when analyzed using longitudinal modeling (S1 Text, S1 Fig and S5 Table). There were no significant effects of condition at any timepoint for the tense or relaxation subscales.

#### Cognitive Interference

**Fig 6:**
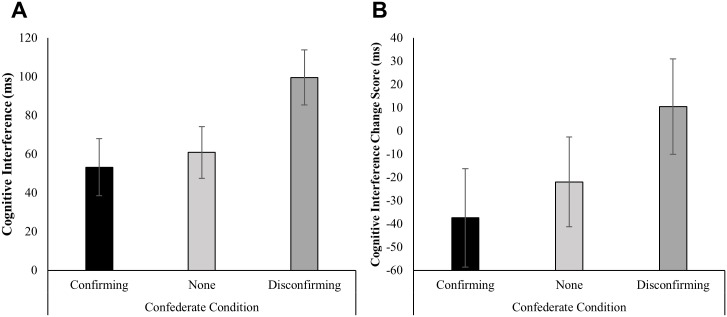
Effects of condition on cognitive interference. Effects are measured as the difference in milliseconds in the time taken to respond to incongruent versus congruent items on a Stroop Task. Error bars represent +/- 1 SE. **(A)** Cognitive interference on the Stroop Task given during minute 25–27, controlling for cognitive interference on the Stroop Task given during minute 4–6, as a function of condition. **(B)** Raw change scores between post- (minute 25–27) and pre-consumption (minute 4–6) levels of cognitive interference, as a function of condition. Adjusted means were obtained from single-level linear regression. *N* = 95.

As predicted, cognitive interference on the Stroop Task was significantly lower in the confirming confederate condition (M_adj_ = 53.27) than in the disconfirming confederate condition (M_adj_ = 99.59), *B* = -46.32, *t*(91) = 2.27, *P* = 0.026, *d* = 0.56. Interference in the disconfirming confederate condition was also higher than interference in the no confederate condition, an effect that was marginally significant (M_adj_ = 60.92), *B* = 38.67, *t*(91) = 1.98, *p* = 0.051, *d* = 0.46. By contrast, interference in the confirming confederate condition was no different than interference in the no confederate condition, *B* = -7.65, *t*(91) = -0.38, *p* = 0.701, *d* = -0.09. Raw differences in the changes in subjective alertness from baseline to post consumption by condition are presented in Fig 6. The full regression models for this outcome are given in S6 Table.

#### Product Endorsement

**Fig 7:**
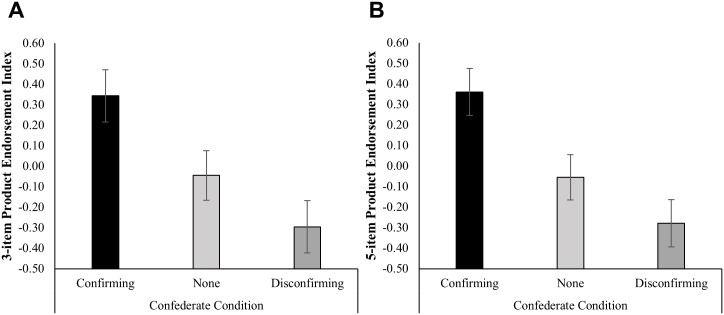
Effect of condition on product endorsement. The y-axis represents an index of product endorsement containing **(A)** only the three items answered at the end of the experiment (minutes 30–32), how much participants think the product should cost, their self-reported probability of buying the product, and their willingness to serve as an ambassador for the product or **(B)** all five items, including the two items answered by a subset of participants one week later, the number of people to whom they positively recommended the product and whether they opted to win some of the product in a lottery. In each case, the index was an average of participants’ available data for each item. Means were obtained from single-level linear regression of the outcome on condition, with no additional covariates. Error bars represent +/- 1 SE. *N* = 99.

For product endorsement, the primary analysis focused on the three-item measure obtained at the end of the experiment (in which subjects indicated how much the product should cost, their probability of buying the product, and their willingness to serve as an ambassador for the product), as all subjects completed these measures. As predicted, participants in the confirming confederate condition (M = 0.34) had higher endorsement of the product relative to the disconfirming confederate condition (M = -0.30), *B* = 0.64, *t*(96) = -3.55, *p* = 0.001, *d* = 0.84. This effect seemed to be driven by the confirming confederate condition. Participants in the confirming confederate condition had significantly higher product endorsement than participants in the no confederate condition (M = -0.04), *B* = 0.39, *t*(95) = 2.21, *p* = 0.030, *d* = 0.51. Participants in the disconfirming confederate condition had lower product endorsement than participants in the no confederate condition, but this difference did not reach significance, *B* = -0.25, *t*(95) = -1.42, *p* = 0.158, *d* = -0.33. The full regression models for this outcome are given in S7 Table. Results were even stronger with the five-item measure, which also included two additional measures of product endorsement completed by approximately half of the subjects one week after the experiment (S7 Table). Differences in the post-consumption levels of product endorsement by condition are presented in Fig 7.

### Caffeine Exposure and Expectancy

Caffeine exposure and caffeine expectancy were tested as covariates in all analyses (see S1 Text and S3–S7 Tables). Caffeine exposure did not significantly affect any outcome. Caffeine expectancy only proved to have a significant effect on subjective alertness at the last measurement (minute 29) (S3 Table).

## Discussion

When participants entered our lab they thought they were drinking the latest in a line of enriched bottled waters. They believed that they were drinking water infused with caffeine, when in fact it was just plain water. Our results demonstrated that the effect of this belief on physiological, psychological and functional alertness was moderated by social influence: participants experienced more subjective alertness, physiological alertness (SBP) and functional alertness (cognitive interference) after consuming the product in the confirming social influence condition than when they consumed the placebo product in the disconfirming social influence condition. Furthermore, the social influence manipulation proved to have effects on product endorsement. Participants in the confirming social influence condition more strongly endorsed the product than participants in the disconfirming social influence condition.

Comparisons between the social influence conditions and the no confederate condition were less consistent but in the expected direction. For subjective alertness, functional alertness (cognitive interference), and physiological alertness (SBP), the disconfirming confederate condition was significantly different than the no confederate condition on the relevant parameters; by contrast, there were no significant differences between the confirming confederate condition and the no confederate condition for these outcomes. With respect to product endorsement, confirming confederate condition participants had significantly higher levels of product endorsement than participants in the no confederate condition, while levels of product endorsement in the disconfirming confederate condition were statistically indistinguishable from those in the no confederate condition. Taken together, these results suggest that whether the effects of social influence are driven more strongly by confirming or disconfirming influence may differ depending on the outcome measure in question. In particular, our results might suggest that product endorsement is moderated by social influence because confirming social influence boosts it whereas product experience (as indicated by the physiological, psychological, and functional effects of the product) is moderated by social influence because disconfirming social influence diminishes it. Of course, any non-significant results may have instead been driven by inadequate statistical power. Therefore, future research with larger samples and in other settings is needed to more deeply understand the different effects of confirming versus disconfirming social influence on various outcome measures.

It is also important to note that participants in the disconfirming confederate condition still reported increases in subjective alertness compared to baseline (even though their increases were significantly lower than the increases found in the confirming confederate condition) while also exhibiting significant reductions in blood pressure and worse cognitive interference as measured by the Stroop task. These patterns may suggest that the effects of disconfirming social influence assert a stronger effect on measures that are less likely to be biased by self-report (i.e., physiological responses). Once again however, any non-significant results may have instead been driven by inadequate statistical power and thus future research with larger samples and in other settings is needed to parse apart the different effects of confirming versus disconfirming social influence on conscious and subconscious indices.

Although future research is needed to more deeply explore the intricacies and nuances of disconfirming and confirming influence on various outcome measures, our results inform and extend the literature on social influence, placebo research and product consumption in two important ways. First, researchers have traditionally struggled to distinguish the effects of social influence on true changes in preference versus mere public compliance [26, 28, 29]. Recent research has informed this debate by employing neurological techniques that suggest that social influence can produce changes in brain areas corresponding to hedonic experience [31]. Our results extend this research and suggest that social influence may also take hold on physiological and functional responses, such as differences in SBP and cognitive interference to a faux stimulant.

Second, our research extends the existing body of research on placebo responses by furthering our understanding of moderating variables that influence the efficacy of placebo effects. The finding that social influence may strengthen placebo response when the expected effect is endorsed (compared to when the effect is denied) may help explain why placebo effects are found in some conditions and not in others [17, 20, 48]: social forces may strengthen or weaken the expected effects. Although the results herein only demonstrate this phenomenon in the context of placebo caffeine, the influence of social modeling on placebo response are likely to be relevant to other domains where placebo effects occur, such as in medicine. Patients are not only influenced by the beliefs and expectations of their doctor, nurse, or caretaker but also by friends, family, and peer-patients who share or have shared the same conditions, medications, and treatments. While much of the science practice of medicine aims to parse out the effects of non-specific or social and psychological factors, the results herein suggest that such factors should not be seen as superfluous but, instead, as potentially active ingredients in clinical care.

Our findings are interesting in light of the increasing popularity of “aquaceuticals” and other products that contain active ingredients while claiming to have effects that are not scientifically supported. If these products are actually producing the claimed effects—even if the cause of the change is driven primarily by the psychosocial context as opposed to the ingredients themselves—does that make the selling of those products and their growing popularity more legitimate? This is a complex question that is beyond the scope of this paper, but our results do remind us that to understand the true effects of any product or substance we must consider carefully the psychosocial setting in which it is consumed.

When reflecting on the legitimacy of marketing claims, it is important to note that the differences in blood pressure in our study did not result from an increase from baseline levels; instead, after the initial placebo effect that began at minute 14, overall blood pressure dropped during the study procedure and this drop was exacerbated in the disconfirming confederate social influence condition but eliminated in the confirming confederate social influence condition. This decline in blood pressure is to be expected given the prolonged sedentary behavior throughout the course of the experiment and is a common occurrence in similar experimental paradigms. However, future research aimed at understanding the effects of the expectation alone would benefit from including a control condition in which participants consumed the same bottled water without the belief that it was caffeinated. While research has already established that placebo caffeine can increase blood pressure response as compared to decaffeinated control [17], our primary aim was to test the moderating role of social influence on physiological responses and the differences in decline are indicative of the impact of social influence on physiological arousal.

Although the results of this study provide important data on the impact of social influence on product experience and endorsement, several questions remain. First, how did the presence of a confederate participant influence physiological or functional responding? Although it is possible that there was an influence from the mere presence of the confederate (Zajonc, 1965), the same presence of a confederate either increased or decreased the effects depending on what the confederate said about the product experience. A more likely mechanism is that what the confederate said about the product experience altered the response expectancies of the participant, which in turn influenced the participant’s physiological and functional responding. In the current study we did not measure the momentary change in expectations because we were concerned that asking participants about their expectations after the social encounter would lead them to question the study guise, and also these expectations would have occurred after random assignment. Thus, more research is needed to thoroughly understand the precise mechanisms (i.e. increased expectancy or other avenues) through which social influence can affect physiological and functional responding. For example, such research might deliberately recruit participants with different levels of baseline caffeine expectancies and assess how they respond to a caffeine placebo product in the presence of a confirming or disconfirming confederate. It is possible that the greatest social influence would occur for high caffeine expectancy participants paired with a confirming confederate and for low caffeine expectancy participants paired with a disconfirming confederate because the confederate would be providing social verification for the participants’ beliefs, thereby strengthening those beliefs [21].

Future research should also examine the effects of consuming the product with another person (or confederate participant) who does not verbally endorse or deny the effect of the product to help disentangle the effects of the social information versus social presence alone. It may be the case that just consuming the product in the presence of another could introduce an element of social influence (i.e., the participant might look over to see what the confederate is doing, and infer from the confederate’s non-verbal behaviors how they are responding to the product). Therefore, future research should also include measures regarding what the target person believes the other is feeling in response to the product.

Second, in today’s world, social influence is often transmitted through technology (via social media, email, etc.). Does technologically-transmitted social influence have the same effect as in-person social influence? There is evidence that the social modeling of a placebo analgesic is effective both when presented in person and when participants view a recording of a participant [49]. However, more work is needed to understand the context in which social influence is likely to have the most potent effects. One question that remains is the degree to which social information is merely providing additional information about the benefits of the product or if there is something inherently unique about a human connection that does or does not provide direct social verification [21].

Third, what is the relative weight of impact from social influence and objective qualities of a product, substance, or experience? We intentionally chose water as our product, rather than something like decaffeinated coffee, in an effort to isolate the effect of social influence from conditioning or other effects that might result from the *smell* of decaffeinated coffee [50]. Future research is needed to understand how social influence interacts with sensory properties to engineer the ultimate impact of a product, medication, or experience.

Finally, what are the boundary conditions of these effects? In the current study we chose to study women only and to match the race and gender of the confederate with the participant to reduce variability and maximize perceived similarity between the participant and the confederate. According to Bandura’s Social Learning Theory, male subjects are more prone to imitate male models and female subjects more prone to imitate female models [51, 52]. Research exploring social modeling on placebo responding has shown mixed effects of gender, with some studies demonstrating greater effects with matched confederates [32, 33], others demonstrating greater effects with male models [53] and still others showing no gender differences in responding or matching affect [34]. The present study is limited in its ability to inform this debate and or make claims outside of female-female interactions.

Though much remains to be explored, our results are intriguing in demonstrating that social influence—working hand in hand with the psychological construction of sensory input—can alter physiological and functional response to a product, in this case changing the experience of, and attitudes toward, simple water.

## Supporting Information

S1 Fig Effects of condition on subjective alertness: longitudinal analyses. (PDF)

S1 File All AquaChargeWater.com website pages. The AquaCharge Energy Water website was designed specifically for the purposes of study by Gibbs Graphics. The website for the fictional product AquaCharge Energy Water was removed from the Internet after the study was complete. All website pages are reprinted under a CC BY license, with permission from Gibbs Graphics, original copyright 2013.
(PDF)

S1 Table Longitudinal regression model for changes in systolic blood pressure from min14 to min24, with time mean-centered at min14. (PDF)

S2 Table Longitudinal regression model for changes in systolic blood pressure from min14 to min24, with time mean-centered at min19. (PDF)

S3 Table Full single-level regression models for average post-consumption levels of subjective alertness. (PDF)

S4 Table Full single-level regression models for post-consumption levels of subjective alertness, separately at min24 and min29. (PDF)

S5 Table Longitudinal regression model for changes in subjective alertness from min13 to min29. (PDF)

S6 Table Full single-level regression models for post-consumption levels of cognitive interference. (PDF)

S7 Table Full single-level regression models for post-consumption levels of product endorsement. (PDF)

S1 Text Additional information on measures and model fitting. (PDF)
