# Supplementary material for: Transforming Water: Social Influence Moderates Psychological, Physiological, and Functional Response to a Placebo Product
Source: PLoS One. 2016 Nov 22;11(11):e0167121. doi: 10.1371/journal.pone.0167121 (PMC5119827; doi:10.1371/journal.pone.0167121)
Supplement: S1 Fig — (PDF) [file pone.0167121.s001.pdf]

S1 Fig. Effects of condition on subjective alertness: longitudinal analyses.

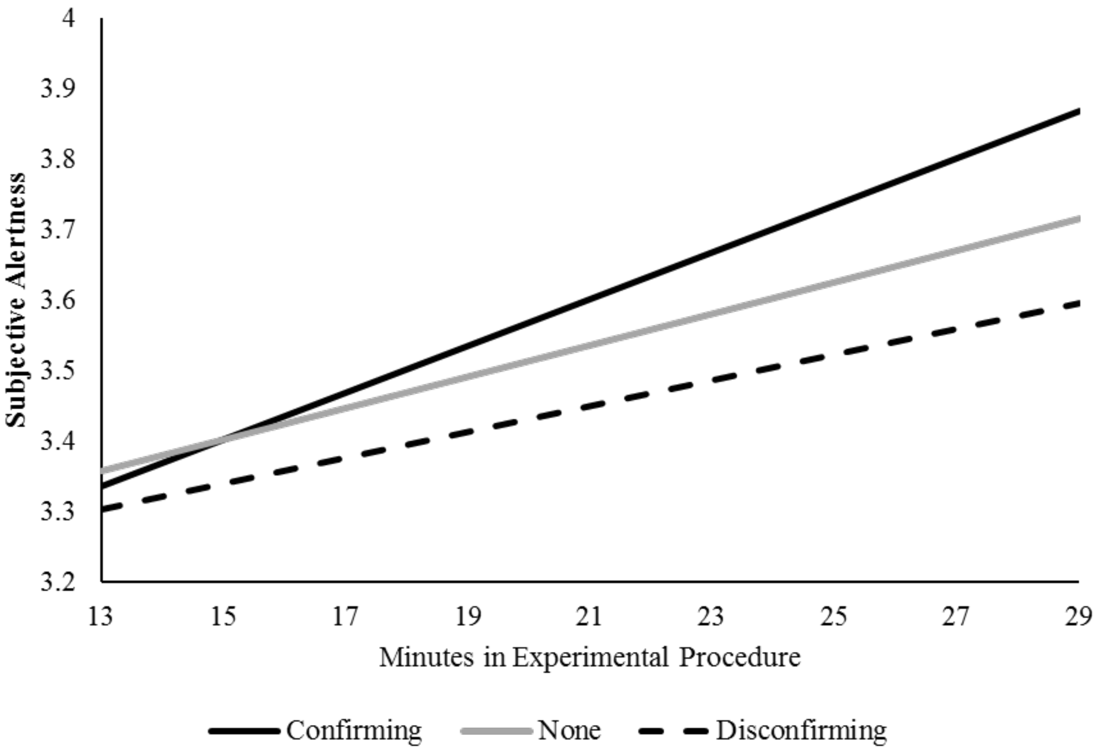

Effects of condition on subjective alertness (on a 1 to 5 scale) over time. Levels of subjective alertness modeling longitudinally using multilevel linear regression. The trajectory reflects measurements taken at three timepoints, at minute 13, before consumption of the AquaCharge product, and at two timepoints post consumption, minutes 24 and 29. Level-1 predictors were initial levels of subjective alertness at minute 13, and the minute by minute rate of change from minute 13 to minute 29. Condition, body mass index, and caffeine expectancy measured prior to the experiment were Level-2 predictors of each Level-1 parameter. For the full longitudinal models from which these means are derived, see S5 Table. N=95.
