# Supplementary material for: Transforming Water: Social Influence Moderates Psychological, Physiological, and Functional Response to a Placebo Product
Source: PLoS One. 2016 Nov 22;11(11):e0167121. doi: 10.1371/journal.pone.0167121 (PMC5119827; doi:10.1371/journal.pone.0167121)
Supplement: S1 File — The AquaCharge Energy Water website was designed specifically for the purposes of study by Gibbs Graphics. The website for the fictional product AquaCharge Energy Water was removed from the Internet after the study was complete. All website pages are reprinted under a CC BY license, with permission from Gibbs Graphics, original copyright 2013. (PDF) [file pone.0167121.s002.pdf]

# AQUACharge

The Buzz   The Product   Get Involved   Who We Are   Contact   Orders

## Have a presentation? Exam? Final? Long drive? Meeting? Marathon to run? Just need to stay awake?

*Get Charged on AquaCharge.*

Caffeine has been shown to improve memory, decrease fatigue, and improve mental functioning. Studies also suggest that consuming caffeine before a workout can result in more endurance, higher performance, less fatigue and quicker recovery times. AquaCharge allows you to get your energy the natural way, with caffeine that is extracted from coffee beans and infused into natural spring water. AquaCharge keeps you feeling refreshed, energized, and ready to go without the calories, without the carbs, and without the chemicals typically used in energy drinks.

***Get Charged. AquaCharged.***

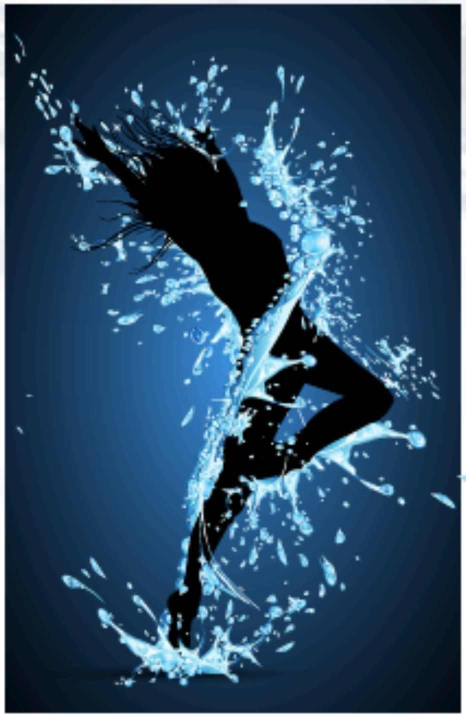

*Get Charged • No Calories • No Chemicals • No Carbs • Caffeine Infused • Natural Spring Water*

Aqua Charge Energy Water, Inc
Email: info@aquachargewater.com
New York, NY

AQUACharge

The Buzz The Product Get Involved Who We Are Contact Orders

## How does AquaCharge compare to other bottled waters and caffeinated drinks?

Other caffeinated drinks use ingredients like high-fructose corn syrup, benzoic acid, and taurine. AquaCharge is made from natural spring water infused with up to 200 mg of caffeine extracted from coffee beans, a process with which is tasteless and odorless.

In other words, AquaCharge is the answer if you want to hydrate and energize in a more natural fashion. Not only does AquaCharge have no chemicals or additives, it has zero calories, zero carbs and a consistent tasteless caffeine boost to keep you charged and hydrated all day long!

AquaCharge comes in three sizes so you can pick the amount of caffeine you need to get your work done. Choose wisely.

The smallest is equivalent to approximately 1 large cup of coffee. Our super AquaCharge has 200 mg of caffeine, enough to really get you charged up.

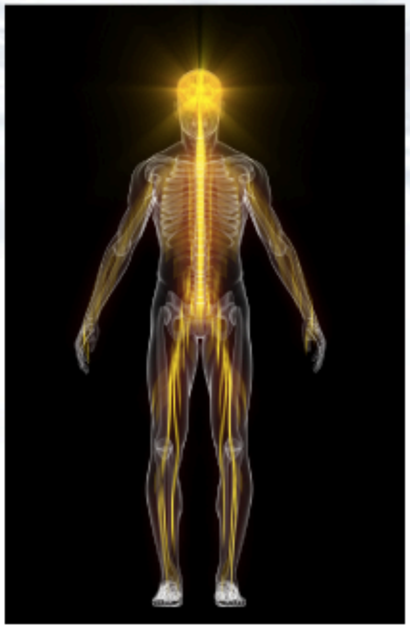

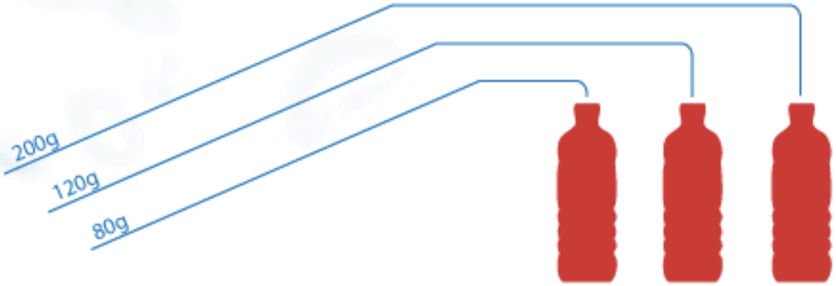


*Get Charged • No Calories • No Chemicals • No Carbs • Caffeine Infused • Natural Spring Water*

Aqua Charge Energy Water, Inc
Email: info@aquachargewater.com
New York, NY

The Buzz The Product Get Involved Who We Are Contact Orders

Want to get AquaCharge in a store near you or just want to show your support? Well, now you can!

## Become a College Ambassador

Need a boost as you prepare for finals, exercise or to stay awake in class? Realize that you are looking for a healthier alternative to the carbs, sugars, calories and chemicals found in other energy drinks? Recognize that the students around you could use the same boost? Bring AquaCharge to your campus by being a College Ambassador.

## Get a resume booster by working with a national brand

The owners of AquaCharge are looking for some dedicated, hardworking and enthusiastic individuals to help take the company to the next level. Whether you are a distributor, store owner, a sales rep, or just charged up on AquaCharge, we want you. Let's do this together!

## Get AquaCharge on or near your campus

If you are not interested in being a College Ambassador but still want to help get the word out, there may be opportunities to represent the brand to retail outlets, convenience and grocery stores around campus.

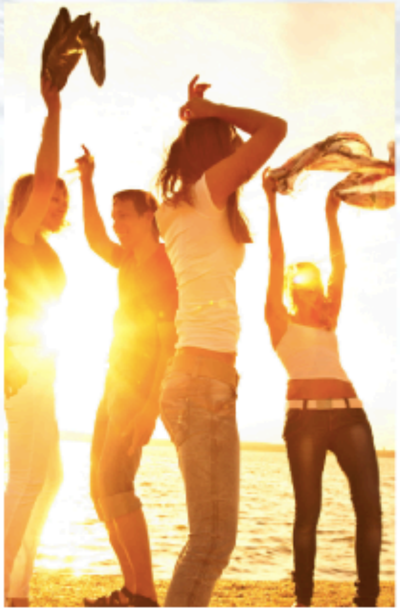

**First Name**

**Last Name**

**E-mail**

## "We couldn't find what we wanted, so we invented it ourselves."

AquaCharge was started in the wee hours at Columbia University's Butler library when two students, Jenny and James, were burning the midnight oil cramming for exams and finishing final papers. Jenny confessed she loved the taste of coffee and the boost it gave her but hated the acidity - and the dread of yellow teeth. James, a Monster and Red Bull fanatic (he had already had three that night), shared that he was sick of the empty calories and chemicals that his body could do without. What was missing was a simple, healthy way to get charged up without all the crap. The idea for AquaCharge was born! From there, Jenny and James brought on additional partners with skills and expertise, including Christine a nationally ranked marathon runner, and Adrianne a burgeoning entrepreneur at Columbia Business School, to create the highest quality and most nutritious energy drink on the market. We hope you enjoy it too.

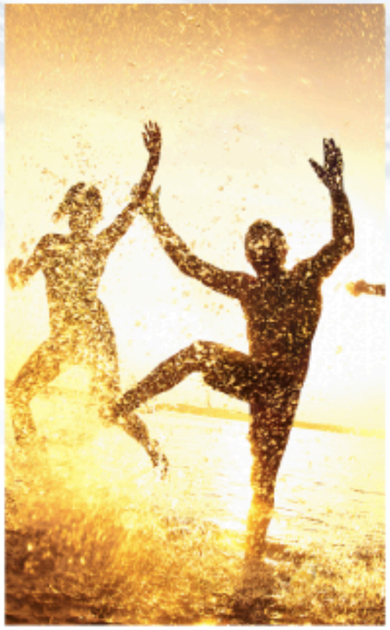

## *Get Charged!*

No calories. No chemicals. No carbs.



Aqua Charge Energy Water, Inc
Email: info@aquachargewater.com
New York, NY

# AQUACharge



At AquaCharge we always want to hear from customers who wish to share their AquaCharge success stories. We will also gladly respond to any inquiries and orders.

AquaCharge Energy Water, Inc.
Email: info@aquachargewater.com
New York, NY

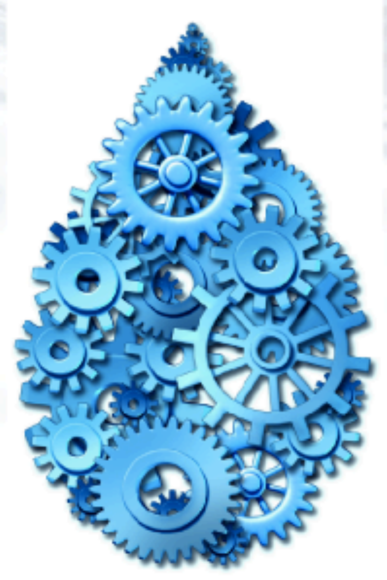

**Your name ***

**Your e-mail address ***

**Subject ***

**Message ***

Send message

*Get Charged • No Calories • No Chemicals • No Carbs • Caffeine Infused • Natural Spring Water*

## Coming Soon

AquaCharge is not yet available in stores. If you wish to purchase AquaCharge, please email us at info@aquachargewater.com and we can arrange a shipment.

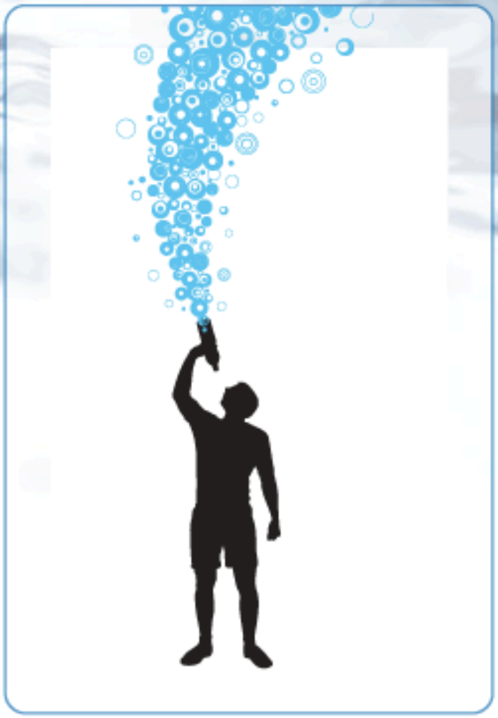

*Get Charged • No Calories • No Chemicals • No Carbs • Caffeine Infused • Natural Spring Water*

Aqua Charge Energy Water, Inc
Email: info@aquachargewater.com
New York, NY
