# Supplementary material for: Transforming Water: Social Influence Moderates Psychological, Physiological, and Functional Response to a Placebo Product
Source: PLoS One. 2016 Nov 22;11(11):e0167121. doi: 10.1371/journal.pone.0167121 (PMC5119827; doi:10.1371/journal.pone.0167121)
Supplement: S1 Table — (PDF) [file pone.0167121.s003.pdf]

S1 Table. Longitudinal regression model for changes in systolic blood pressure from min14 to min24, with time mean-centered at min14.

	Parameter (γ)	90% Winsorized Method				3 SD Outlier Method			
		Orthogonal		Dummy		Orthogonal		Dummy	
		B	SE	*B*	SE	*B*	SE	*B*	SE
**Fixed Effects**									
Level-1 Intercept (π_{0i})									
Intercept	00	117.99***	0.91	116.97***	1.59	117.96***	1.00	116.83***	1.74
Condition: Confederate vs No Confederate	01	1.02	1.29			1.13	1.42		
Condition: Disconfirming vs Confirming	02	-2.31	2.23			-2.01	2.45		
Condition: Confirming vs No Confederate	01			2.68	2.24			2.69	2.47
Condition: Disconfirming vs No Confederate	02			0.37	2.23			0.69	2.43
Average Baseline Systolic Blood Pressure	03	0.96***	0.07	0.96***	0.07	0.99***	0.07	0.99***	0.07
Slope from Minute 14-19 (π_{1i})									
Intercept	10	0.16	0.20	0.13	0.36	0.01	0.23	-0.01	0.39
Condition: Confederate vs No Confederate	11	0.03	0.29			0.02	0.32		
Condition: Disconfirming vs Confirming	12	1.03*	0.50			1.17*	0.55		
Condition: Confirming vs No Confederate	11			-0.48	0.50			-0.56	0.56
Condition: Disconfirming vs No Confederate	12			0.56	0.50			0.61	0.55
Average Baseline Systolic Blood Pressure	13	0.02	0.02	0.02	0.02	0.01	0.02	0.01	0.02
Shift in Slope at Minute 19 (π_{2i})									
Intercept	20	-0.58*	0.28	-0.1	0.49	-0.52~	0.31	-0.05	0.54
Condition: Confederate vs No Confederate	21	-0.47	0.40			-0.47	0.44		
Condition: Disconfirming vs Confirming	22	-1.93**	0.69			-2.14**	0.75		
Condition: Confirming vs No Confederate	21			0.26	0.70			0.37	0.76
Condition: Disconfirming vs No Confederate	22			-1.67*	0.69			-1.77*	0.75
Average Baseline Systolic Blood Pressure	23	-0.02	0.02	-0.02	0.02	-0.01	0.02	-0.01	0.02

**Variance Components**					
Level-1					
within-person	σ_{ε}^2	53.3***	53.3***	61.85***	61.85***
Level-2					
In level-1 intercept	σ_0^2	51.38***	51.38***	62.2***	62.2***
In slope (min 14-19)	σ_1^2	1.57***	1.57***	1.99***	1.99***
Covariance	σ_{01}	-4.98***	-4.98***	-7.07***	-7.07***
**Goodness-of-fit**					
Deviance		7462.2	7462.2	7462.292	7462.292
AIC		7494.2	7494.2	7494.292	7494.292
BIC		7535.1	7535.1	7534.985	7534.985

Note. ~p≤0.10, *p≤0.05, **p≤0.01, ***p≤0.001. The table presents the regression coefficients, standard errors, and goodness-of-fit statistics for the best-fitting longitudinal model for predicting levels of systolic blood pressure (SBP) in each minute of the experiment from minute 14, when participants began consuming the product, to minute 24, directly before the second Stroop Task. To obtain these coefficients, we used multilevel linear regression, in which measurement occasions were nested in participants. Using likelihood ratio tests of a larger set of nested models (not shown), we selected as the final model the model with the lowest overall deviance. Time was measured in minutes and mean-centered at minute 14. The slope, or rate of change, is the change in mmHg per minute. The predictors in the left column are Level-1 individual growth parameters, each of which is predicted by the Level-2 predictors indented directly below it. π_{0i} is the level of SBP at minute 14. π_{1i} is the slope of SBP from minute 14 to minute 19, and π_{2i} is a time-varying predictor that represents the shift in slope during minute 19-24 relative to the slope during minute 14-19. Each Level-1 parameter was allowed to differ as a function of condition, controlling for (grand mean centered) SBP during the baseline period, from minute 1 to 13. We fit models in which condition was coded either with dummy codes or with orthogonal contrast codes (each yielded equivalent goodness of fit statistics). Because the machine used to measure blood pressure sometimes produced outliers, we addressed this using a 90% winsorization within each minute (left panel) or by dropping observations that lay beyond 3 SD of the mean in either direction for each minute (right panel). Each model contained four random effects, as shown. We report results from the left panel in the main text. *N*=95. Deviance=-2 * log likelihood. AIC =Akaike Information Criterion. BIC=Bayesian Information Criterion.
