# Supplementary material for: Transforming Water: Social Influence Moderates Psychological, Physiological, and Functional Response to a Placebo Product
Source: PLoS One. 2016 Nov 22;11(11):e0167121. doi: 10.1371/journal.pone.0167121 (PMC5119827; doi:10.1371/journal.pone.0167121)
Supplement: S2 Table — (PDF) [file pone.0167121.s004.pdf]

S2 Table. Longitudinal regression model for changes in systolic blood pressure from min14 to min24, with time mean-centered at min19.

	Parameter (γ)	90% Winsorized Method				3 SD Outlier Method			
		Orthogonal		Dummy		Orthogonal		Dummy	
		B	SE	*B*	SE	*B*	SE	*B*	SE
**Fixed Effects**									
Level-1 Intercept (π_{0i})									
Intercept	00	118.77***	0.79	117.62***	1.39	118.02***	0.82	116.8***	1.44
Condition: Confederate vs No Confederate	01	1.15	1.13			1.21	1.17		
Condition: Disconfirming vs Confirming	02	2.86	1.95			3.83~	2.02		
Condition: Confirming vs No Confederate	01			0.29	1.96			-0.09	2.04
Condition: Disconfirming vs No Confederate	02			3.15	1.95			3.73~	2.01
Average Baseline Systolic Blood Pressure	03	1.06***	0.06	1.06***	0.06	1.06***	0.06	1.06***	0.06
Slope from Minute 19-24 (π_{1i})									
Intercept	10	-0.42*	0.2	0.03	0.36	-0.51*	0.23	-0.06	0.40
Condition: Confederate vs No Confederate	11	-0.45	0.29			-0.45	0.32		
Condition: Disconfirming vs Confirming	12	-0.9~	0.5			-0.97~	0.55		
Condition: Confirming vs No Confederate	11			-0.22	0.5			-0.19	0.56
Condition: Disconfirming vs No Confederate	12			-1.12*	0.5			-1.16*	0.55
Average Baseline Systolic Blood Pressure	13	0	0.02	0	0.02	0	0.02	0	0.02
Shift in Slope at Minute 14 (π_{2i})									
Intercept	20	0.58*	0.28	0.1	0.49	0.52~	0.31	0.05	0.54
Condition: Confederate vs No Confederate	21	0.47	0.4			0.47	0.44		
Condition: Disconfirming vs Confirming	22	1.93**	0.69			2.14**	0.75		
Condition: Confirming vs No Confederate	21			-0.26	0.7			-0.37	0.76
Condition: Disconfirming vs No Confederate	22			1.67*	0.69			1.77*	0.75
Average Baseline Systolic Blood Pressure	23	0.02	0.02	0.02	0.02	0.01	0.02	0.01	0.02
**Variance Components**									

Level-1					
within-person	σ_ε^2	53.3***	53.3***	61.85***	61.85***
Level-2					
In level-1 intercept	σ_0^2	40.91***	40.91***	41.24***	41.24***
In slope (min 19-24)	σ_1^2	1.57***	1.57***	1.99***	1.99***
Covariance	σ_{01}	2.88**	2.88**	2.88**	2.88**
**Goodness-of-fit**					
Deviance		7462.214	7462.214	7462.292	7462.292
AIC		7494.214	7494.214	7494.292	7494.292
BIC		7535.076	7535.076	7534.985	7534.985

Note. ~p≤0.10, *p≤0.05, **p≤0.01, ***p≤0.001. The table presents the regression coefficients, standard errors, and goodness-of-fit statistics for the same longitudinal model displayed in S1 Table, except time is mean-centered at minute 19 rather than minute 14, to better illuminate the slopes in the post shared reality period after the confederate speaks in the social influence conditions. This set of models has equivalent goodness-of-fit statistics as the models in S1 Table but the parameters have different interpretations. π_{0i} is now the level of SBP at minute 19. π_{1i} is the slope of SBP from minute 19 to minute 24, and π_{2i} is a time-varying predictor that represents the shift in slope during minute 14-19 relative to the slope during minute 19-24. Each Level-1 parameter was allowed to differ as a function of condition, controlling for (grand mean centered) SBP during the baseline period, from minute 1 to 13. As before, we fit models in which condition was coded either with dummy codes or with orthogonal contrast codes (each yielded equivalent goodness of fit statistics) and using both methods for addressing outliers, a 90% winsorization within each minute (left panel) or dropping observations that lay beyond 3 SD of the mean in either direction for each minute (right panel). Each model contained four random effects, as shown. We report results from the left panel in the main text. *N*=95. Deviance=-2 * log likelihood. AIC =Akaike Information Criterion. BIC=Bayesian Information Criterion.
