# Supplementary material for: Transforming Water: Social Influence Moderates Psychological, Physiological, and Functional Response to a Placebo Product
Source: PLoS One. 2016 Nov 22;11(11):e0167121. doi: 10.1371/journal.pone.0167121 (PMC5119827; doi:10.1371/journal.pone.0167121)
Supplement: S3 Table — (PDF) [file pone.0167121.s005.pdf]

S3 Table. Full single-level regression models for average post-consumption levels of subjective alertness.

	Subjective Alertness			
	Minute 24 and 29 Average			
	B1	B2	B3	B4
Dummy-Coded Condition				
Confirming vs No Confederate	0.11	0.14	0.15	0.15
	(0.14)	(0.10)	(0.10)	(0.10)
Disconfirming vs No Confederate	-0.2	-0.18~	-0.1	-0.1
	(0.14)	(0.10)	(0.10)	(0.10)
Intercept	3.7***	3.68***	3.65***	3.66***
	(0.10)	(0.07)	(0.07)	(0.07)
Orthogonally-Coded Condition				
Confederate vs No Confederate	-0.03	-0.01	0.02	0.02
	(0.08)	(0.06)	(0.06)	(0.06)
Disconfirming vs Confirming	-0.31*	-0.32**	-0.25*	-0.24*
	(0.14)	(0.10)	(0.10)	(0.10)
Intercept	3.67***	3.67***	3.67***	3.67***
	(0.06)	(0.04)	(0.04)	(0.04)
Baseline Covariates				
Subjective Alertness (Min. 13)		0.6***	0.62***	0.62***
		(0.06)	(0.06)	(0.06)
Caffeine Expectancy			0.12**	0.11*
			(0.04)	(0.04)
Caffeine Exposure				0.01
				(0.02)
Goodness-of-fit				
R-Squared	0.05	0.51	0.54	0.54
Change in R-Squared	0.05~	0.46***	0.03**	0
N	97	97	97	97

Note. ~p≤0.10, *p≤0.05, **p≤0.01, ***p≤0.001. Regression coefficients, standard errors, and associated goodness-of-fit statistics predicting average post-consumption levels of subjective alertness, averaged across levels at minute 24 and 29. Standard errors are in parentheses directly below the relevant regression coefficient. B1 to B4 represent blocks in a stepwise single-level linear regression. B1 represents the effect of adding condition, B2 represents the effect of adding pre-consumption levels of subjective alertness, B3 represents the effect of adding baseline caffeine expectancy, and B4 represents the effect of adding baseline caffeine exposure. Stepwise regression was conduced twice, using either dummy-coded condition or orthogonally-coded condition (either coding yields equivalent goodness-of-fit statistics). In each case condition was coded with two variables: contrast 1 (dummy: 0,+1,0; orthogonal: -1; +0.5; +0.5) and contrast 2 (dummy: 0,0,+1; orthogonal: 0; -0.5; +0.5), for the no confederate, confirming, and disconfirming confederate conditions respectively. With dummy coded condition, the intercept represents the average adjusted level of subjective alertness for the no confederate condition. With orthogonally coded condition, the intercept represents the average adjusted level of subjective alertness for all participants. B2 is the block reported in the main text.
