# Supplementary material for: Transforming Water: Social Influence Moderates Psychological, Physiological, and Functional Response to a Placebo Product
Source: PLoS One. 2016 Nov 22;11(11):e0167121. doi: 10.1371/journal.pone.0167121 (PMC5119827; doi:10.1371/journal.pone.0167121)
Supplement: S4 Table — (PDF) [file pone.0167121.s006.pdf]

S4 Table. Full single-level regression models for post-consumption levels of subjective alertness, separately at min24 and min29.

	Subjective Alertness							
	Minute 24				Minute 29			
	B1	B2	B3	B4	B1	B2	B3	B4
Dummy-Coded Condition								
Confirming vs No Confederate	0.14	0.17	0.17	0.17	0.09	0.11	0.13	0.12
	(0.15)	(0.11)	(0.11)	(0.11)	(0.15)	(0.12)	(0.11)	(0.11)
Disconfirming vs No Confederate	-0.2	-0.17	-0.14	-0.14	-0.15	-0.15	-0.03	-0.04
	(0.15)	(0.11)	(0.12)	(0.12)	(0.15)	(0.12)	(0.12)	(0.12)
Intercept	3.62***	3.6***	3.59***	3.59***	3.78***	3.77***	3.72***	3.73***
	(0.10)	(0.08)	(0.08)	(0.08)	(0.10)	(0.08)	(0.08)	(0.08)
Orthogonally-Coded Condition								
Confederate vs No Confederate	-0.02	0	0.01	0.01	-0.02	-0.01	0.03	0.03
	(0.09)	(0.06)	(0.07)	(0.07)	(0.09)	(0.07)	(0.07)	(0.07)
Disconfirming vs Confirming	-0.34*	-0.34**	-0.31**	-0.31**	-0.23	-0.27*	-0.16	-0.16
	(0.15)	(0.11)	(0.12)	(0.12)	(0.15)	(0.12)	(0.12)	(0.12)
Intercept	3.6***	3.6***	3.6***	3.6***	3.76***	3.75***	3.75***	3.75***
	(0.06)	(0.05)	(0.05)	(0.05)	(0.06)	(0.05)	(0.05)	(0.05)
Baseline Covariates								
Subjective Alertness (Min. 13)		0.6***	0.61***	0.61***		0.57***	0.6***	0.61***
		(0.07)	(0.07)	(0.07)		(0.08)	(0.07)	(0.07)
Caffeine Expectancy			0.05	0.05			0.18***	0.17***
			(0.05)	(0.05)			(0.05)	(0.05)
Caffeine Exposure				0				0.02
				(0.02)				(0.02)
Goodness-of-fit								
R-Squared	0.05	0.47	0.47	0.47	0.02	0.38	0.46	0.47
Change in R-Squared	0.05~	0.42***	0.01	0	0.02	0.36***	0.08***	0.01
N	96	96	96	96	96	96	96	96

Note. ~p≤0.10, *p≤0.05, **p≤0.01, ***p≤0.001. Regression coefficients, standard errors, and associated goodness-of-fit statistics predicting post-consumption levels of subjective alertness, separately at minute 24 (left panel) and minute 29 (right panel). Standard errors are in parentheses directly below the relevant regression coefficient. B1 to B4 represent blocks in a stepwise single-level linear regression, conducted separately for

each timepoint. B1 represents the effect of adding condition, B2 represents the effect of adding pre-consumption levels of subjective alertness, B3 represents the effect of adding baseline caffeine expectancy, and B4 represents the effect of adding baseline caffeine exposure. For each timepoint, stepwise regression was conduced twice, using either dummy-coded condition or orthogonally-coded condition (either coding yields equivalent goodness-of-fit statistics). In each case condition was coded with two variables: contrast 1 (dummy: 0,+1,0; orthogonal: -1; +0.5; +0.5) and contrast 2 (dummy: 0,0,+1; orthogonal: 0; -0.5; +0.5), for the no confederate, confirming, and disconfirming confederate conditions respectively. With dummy coded condition, the intercept represents the average adjusted level of subjective alertness for the no confederate condition. With orthogonally coded condition, the intercept represents the average adjusted level of subjective alertness for all participants.
