# Supplementary material for: Transforming Water: Social Influence Moderates Psychological, Physiological, and Functional Response to a Placebo Product
Source: PLoS One. 2016 Nov 22;11(11):e0167121. doi: 10.1371/journal.pone.0167121 (PMC5119827; doi:10.1371/journal.pone.0167121)
Supplement: S5 Table — (PDF) [file pone.0167121.s007.pdf]

S5 Table. Longitudinal regression model for changes in subjective alertness from min13 to min29.

		Orthogonal				Dummy			
		Basic		Final		Basic		Final	
	Parameter (γ)	B	SE	B	SE	B	SE	B	SE
**Fixed Effects**									
Level-1 Intercept (π_{0i})									
Intercept	00	3.328***	(0.063)	3.331***	(0.064)	3.339***	(0.106)	3.357***	(0.109)
Condition: Confederate vs No Confederate	01	-0.011	(0.088)	-0.025	(0.091)				
Condition: Disconfirming vs Confirming	02	-0.005	(0.157)	-0.032	(0.166)				
Condition: Confirming vs No Confederate	01					-0.014	(0.154)	-0.022	(0.154)
Condition: Disconfirming vs No Confederate	02					-0.018	(0.154)	-0.054	(0.166)
Body Mass Index	03			0.019	(0.017)			0.019	(0.017)
Caffeine Expectancy	04			-0.058	(0.071)			-0.058	(0.071)
Slope from Minute 13-29 (π_{1i})									
Intercept	10	0.026***	(0.003)	0.025***	(0.003)	0.027***	(0.006)	0.022***	(0.005)
Condition: Confederate vs No Confederate	11	-0.001	(0.005)	0.002	(0.004)				
Condition: Disconfirming vs Confirming	12	-0.021*	(0.008)	-0.015~	(0.008)				
Condition: Confirming vs No Confederate	11					0.008	(0.008)	0.011	(0.007)
Condition: Disconfirming vs No Confederate	12					-0.012	(0.008)	-0.004	(0.008)
Body Mass Index	13			0.001	(0.001)			0.001	(0.001)
Caffeine Expectancy	14			0.013***	(0.003)			0.013***	(0.003)
**Variance Components**									
Level-1									
within-person	σ_{ε}^{2}	0.099***		0.074***		0.099***		0.074***	
Level-2									
In level-1 intercept	σ_{0}^{2}	0.303***		0.321***		0.303***		0.321***	
In rate of change (slope)	σ_{1}^{2}	0*		0*		0*		0*	
Covariance	σ_{01}	-0.005~		-0.005*		-0.005~		-0.005*	
**Goodness-of-fit**									

Deviance	410.31	342.33	410.31	342.33
AIC	430.31	370.33	430.31	370.33
BIC	456.26	406.23	456.26	406.23
N	99	96	99	96

Note. ~p≤0.10, *p≤0.05, **p≤0.01, ***p≤0.001. The table presents the regression coefficients, standard errors, and goodness-of-fit statistics for the best-fitting longitudinal model for predicting levels of subjective alertness (SBP) at three timepoints during the experiment, at minute 13, 24, and 29. To obtain these coefficients, we used multilevel linear regression, in which measurement occasions were nested in participants. Using likelihood ratio tests, we selected as the final model the model with the lowest overall deviance, but also show the basic condition only model side by side for comparison. Time was measured in minutes and mean-centered at minute 13. The slope, or rate of change, is the change in levels of subjective alertness per minute, assuming linear change. The predictors in the left column are Level-1 individual growth parameters, each of which is predicted by the Level-2 predictors indented directly below it. π_{0i} is the level of subjective alertness at minute 13, the baseline measurement. π_{1i} is the slope of subjective alertness from minute 13 to minute 29 (the full change during this period can be obtained by multiplying the coefficient by 16, the total number of minutes in the analysis period). Each Level-1 parameter was allowed to differ as a function of condition, and the final model also controlled for (grand mean centered) body mass index and caffeine expectancy at Level 2. We fit models in which condition was coded either with dummy codes or with orthogonal contrast codes (each yielded equivalent goodness of fit statistics). Each model contained four random effects, as shown. In the main text we report a cross-sectional version of these models (see Table S1). Deviance=-2 * log likelihood. AIC =Akaike Information Criterion. BIC=Bayesian Information Criterion
