# Supplementary material for: Transforming Water: Social Influence Moderates Psychological, Physiological, and Functional Response to a Placebo Product
Source: PLoS One. 2016 Nov 22;11(11):e0167121. doi: 10.1371/journal.pone.0167121 (PMC5119827; doi:10.1371/journal.pone.0167121)
Supplement: S6 Table — (PDF) [file pone.0167121.s008.pdf]

S6 Table. Full single-level regression models for post-consumption levels of cognitive interference.

	Cognitive Interference (Minute 25-26)			
	B1	B2	B3	B4
Dummy-Coded Condition				
Confirming vs No Confederate	-3.49	-6.05	-6.08	-6.67
	(20.81)	(20.16)	(20.27)	(20.42)
Disconfirming vs No Confederate	41.14*	38.77~	37.84~	37.75~
	(20.62)	(19.97)	(21.02)	(21.12)
Intercept	57.68***	59.32***	59.64***	59.9***
	(14.12)	(13.68)	(13.91)	(13.99)
Orthogonally-Coded Condition				
Confederate vs No Confederate	12.55	10.91	10.59	10.36
	(11.82)	(11.45)	(11.71)	(11.78)
Disconfirming vs Confirming	44.63*	44.82*	43.92*	44.42*
	(21.44)	(20.75)	(21.70)	(21.83)
Intercept	70.23***	70.23***	70.23***	70.26***
	(8.56)	(8.28)	(8.33)	(8.37)
Baseline Covariates				
Cognitive Interference (Min. 4-5)		0.21**	0.21**	0.21**
		(0.08)	(0.08)	(0.08)
Caffeine Expectancy			-1.35	-2.02
			(8.97)	(9.15)
Caffeine Exposure				1.43
				(3.38)
Goodness-of-fit				
R-Squared	0.06	0.13	0.13	0.13
Change in R-Squared	0.06~	0.07**	0	0
N	93	93	93	93

Note. ~p≤0.10, *p≤0.05, **p≤0.01, ***p≤0.001. Regression coefficients, standard errors, and associated goodness-of-fit statistics predicting post-consumption levels of cognitive interference, as measured on a Stroop Task given from minutes 25-27. Standard errors are in parentheses directly below the relevant regression coefficient. B1 to B4 represent blocks in a stepwise single-level linear regression. B1 represents the effect of adding condition, B2 represents the effect of adding pre-consumption levels of cognitive interference (during an initial Stroop Task at minutes 4-6), B3 represents the effect of adding baseline caffeine expectancy, and B4 represents the effect of adding baseline caffeine exposure. Stepwise regression was conduced twice, using either dummy-coded condition or orthogonally-coded condition (either coding yields equivalent goodness-of-fit statistics). In each case condition was coded with two variables: contrast 1 (dummy: 0,+1,0; orthogonal: -1; +0.5; +0.5) and contrast 2 (dummy: 0,0,+1; orthogonal: 0; -0.5; +0.5), for the no confederate, confirming, and disconfirming confederate conditions respectively. With dummy coded condition, the intercept represents the average adjusted level of cognitive interference for the no confederate condition. With orthogonally coded condition, the intercept represents the average adjusted level of cognitive interference for all participants. B2 is the block reported in the main text.
