# Supplementary material for: Transforming Water: Social Influence Moderates Psychological, Physiological, and Functional Response to a Placebo Product
Source: PLoS One. 2016 Nov 22;11(11):e0167121. doi: 10.1371/journal.pone.0167121 (PMC5119827; doi:10.1371/journal.pone.0167121)
Supplement: S7 Table — (PDF) [file pone.0167121.s009.pdf]

**S7 Table**. **Full single-level regression models for post-consumption levels of product endorsement.**

| | Product Endorsement | | | | | | | |
|---|---|---|---|---|---|---|---|---|
| | 3-Item Composite | | | | 5-Item Composite | | | |
| | B1 | B2 | B3 | B4 | B1 | B2 | B3 | B4 |
| Dummy-Coded Condition | | | | | | | | |
| Confirming vs No Confederate | 0.39* | 0.39* | 0.4* | 0.41* | 0.41** | 0.41* | 0.41** | 0.42** |
| | (0.18) | (0.18) | (0.18) | (0.18) | (0.16) | (0.16) | (0.16) | (0.16) |
| Disconfirming vs No Confederate | -0.24 | -0.24 | -0.17 | -0.16 | -0.21 | -0.22 | -0.16 | -0.15 |
| | (0.18) | (0.18) | (0.19) | (0.19) | (0.16) | (0.16) | (0.17) | (0.17) |
| Intercept | -0.04 | -0.04 | -0.07 | -0.08 | -0.05 | -0.05 | -0.07 | -0.08 |
| | (0.12) | (0.13) | (0.13) | (0.13) | (0.11) | (0.11) | (0.11) | (0.12) |
| Orthogonally-Coded Condition | | | | | | | | |
| Confederate vs No Confederate | 0.05 | 0.05 | 0.08 | 0.08 | 0.07 | 0.06 | 0.08 | 0.09 |
| | (0.10) | (0.10) | (0.11) | (0.11) | (0.09) | (0.09) | (0.10) | (0.10) |
| Disconfirming vs Confirming | -0.63*** | -0.63*** | -0.57** | -0.57** | -0.63*** | -0.62*** | -0.57*** | -0.58*** |
| | (0.18) | (0.18) | (0.19) | (0.19) | (0.17) | (0.17) | (0.17) | (0.17) |
| Intercept | 0.01 | 0.01 | 0.01 | 0.01 | 0.01 | 0.01 | 0.01 | 0.01 |
| | (0.07) | (0.07) | (0.07) | (0.07) | (0.07) | (0.07) | (0.07) | (0.07) |
| Baseline Covariates | | | | | | | | |
| Bottled Water Frequency | | 0 | 0 | -0.01 | | 0.01 | 0.01 | 0.01 |
| | | (0.04) | (0.04) | (0.04) | | (0.03) | (0.03) | (0.03) |
| Caffeine Expectancy | | | 0.1 | 0.11 | | | 0.08 | 0.09 |
| | | | (0.08) | (0.08) | | | (0.07) | (0.07) |
| Caffeine Exposure | | | | -0.03 | | | | -0.02 |
| | | | | (0.03) | | | | (0.03) |
| Goodness-of-fit | | | | | | | | |
| R-Squared | 0.11 | 0.11 | 0.13 | 0.14 | 0.14 | 0.14 | 0.15 | 0.16 |
| Change in R-Squared | 0.11** | 0 | 0.02 | 0.01 | 0.14*** | 0 | 0.01 | 0.01 |
| N | 97 | 97 | 97 | 97 | 97 | 97 | 97 | 97 |

*Note.* ~p≤0.10, *p≤0.05, **p≤0.01, ***p≤0.001. Regression coefficients, standard errors, and associated goodness-of-fit statistics predicting post-consumption levels of product endorsement. The left panel represents results when the composite contains only the three items given during minutes 30 to 32 of the experiment. The right panel represents results when the composite also includes two additional measures of product endorsement given a week after the experiment, to which approximately half of participants responded. Standard errors are in parentheses directly

below the relevant regression coefficient. B1 to B4 represent blocks in a stepwise single-level linear regression. B1 represents the effect of adding condition, B2 represents the effect of adding baseline self-reported frequency of drinking bottled water, B3 represents the effect of adding baseline caffeine expectancy, and B4 represents the effect of adding baseline caffeine exposure. Stepwise regression was conduced twice, using either dummy-coded condition or orthogonally-coded condition (either coding yields equivalent goodness-of-fit statistics). In each case condition was coded with two variables: contrast 1 (dummy: 0,+1,0; orthogonal: -1; +0.5; +0.5) and contrast 2 (dummy: 0,0,+1; orthogonal: 0; -0.5; +0.5), for the no confederate, confirming, and disconfirming confederate conditions respectively. With dummy coded condition, the intercept represents the average adjusted level of product endorsement for the no confederate condition. With orthogonally coded condition, the intercept represents the average adjusted level of product endorsement for all participants. B1 in the left panel is the block reported in the main text
