# Supplementary material for: Transforming Water: Social Influence Moderates Psychological, Physiological, and Functional Response to a Placebo Product
Source: PLoS One. 2016 Nov 22;11(11):e0167121. doi: 10.1371/journal.pone.0167121 (PMC5119827; doi:10.1371/journal.pone.0167121)
Supplement: S1 Text — (PDF) [file pone.0167121.s010.pdf]

S1 Text. Additional information on measures and model fitting.

Baseline Measures

Several baseline measures were obtained prior to the formal experiment as possible covariates:

Caffeine exposure. Caffeine exposure was assessed with a single item, "In a typical week, how many days do you consume coffee or caffeinated energy drinks?" Responses could range from 0 to 7.

Body mass index (BMI). BMI was calculated using participants' self-reported height and weight, as their weight in kg divided by their height in cm squared.

Caffeine expectancy. Caffeine expectancy was assessed with 11 items (e.g., "Caffeine makes me feel more alert"; 1=*very unlikely*, 6=*very likely*). The reliability of the scale was $\propto$=0.92.

Frequency of drinking bottled water. Frequency of drinking bottled water was assessed with one item, "How often do you drink bottled water?". Responses could range from 1=*never* to 7=*daily*.

Ethnicity. Ethnicity could be one of five categories: Black, White, Asian, Indian/Middle Eastern, or Hispanic. For purposes of analysis, it was reduced to a contrast-coded dichotomous variable. Negatively stereotyped ethnicities (Black, Indian/Middle Eastern, Hispanic) were given a value of 1; non-stereotyped ethnicities were given a value of -1.

Random Assignment

To check for random assignment to condition, we regressed the baseline covariates described above on condition, in separate regressions. With one exception, there were no differences in any of the baseline covariates as a function of condition, ts<1.30, *p*s>= 0.20. The exception was caffeine expectancy. Participants in the disconfirming confederate condition had significantly lower levels of caffeine expectancy than participants in the no confederate condition, B=-0.69, $t(94)$=-2.94, p=0.004, or participants in the confirming confederate condition, p=-0.62, $t(94)$=-2.61, p=0.011. Because of this, we tested additional regression models that also controlled for caffeine expectancy, as described below.

Single-Level (Cross-Sectional) Data Analysis and Model Fitting Strategy

We used single-level multiple regression to assess the effect of condition on three primary cross-sectional outcomes: subjective alertness, cognitive interference (functional alertness), and an index of product endorsement, at key timepoints post consumption. To compare the confirming and disconfirming confederate conditions to the no confederate (control) condition, we used two dummy variables, with each assigning a value of 0 to the no confederate condition (0,+1,0; 0,0,+1). To directly compare the confirming confederate and disconfirming confederate conditions, we used orthogonal coding. One variable compared the average of the confederate conditions (each assigned a value of 0.5) to the no confederate condition (-1). The second variable compared the disconfirming (+0.5) to the confirming confederate condition (-0.5); the no confederate condition was set to 0 for this variable. For the two alertness measures, we controlled for the baseline measure of these constructs, after grand-mean centering them. We present effect size in standard deviation units of the raw outcome mean for the full sample.
The final model reported in the main text contains only the effect of condition, and when available, the baseline measure of the outcome. For each model, we also controlled for caffeine

exposure and caffeine expectancy, as shown in S3-S7 Tables. In general, these additional covariates did not change the significance of the focal comparison, the confirming confederate conditionto the disconfirming confederate condition, except for subjective alertness at the final timepoint, minute 29.

Longitudinal Data Analysis and Model Fitting Strategy

For every longitudinal analysis, we fit multilevel models that contained two submodels: a Level-1 submodel that described how the outcome changed over time for a given participant, and Level-2 submodels that predicted key parameters of the Level-1 model as a function of condition. We also included time-varying predictors at Level 1 when these allowed us to test additional hypotheses beyond effects on basic linear slope; these permitted intercepts or slopes to vary by condition as a function of different time periods associated with key inflection points in the experimental procedure. These inflection points included minute 14, when participants first began drinking the beverage; minute 16, the start of the post consumption period; minute 19, when the confederate first spoke in the two confederate conditions, and minute 25, the start of the second Stroop Task.

For each distinct epoch, there could be a shift in either elevation, slope, or both, as a function of condition. To determine the best fitting model, we fit a systematic taxonomy of statistical models with the same predictors for condition at Level 2 but different time-varying predictors at Level 1 (each associated with a different type of discontinuity, for one or more time periods). We selected the final model for each analysis by comparing deviance statistics for nested models (likelihood ratio tests) containing different combinations of time-varying predictors. For systolic blood pressure, we were particularly interested in the time period from minute 14 to minute 24. Through likelihood ratio testing of different models using measurements from this time period, we determined the best-fitting model for the change in this outcome over time was one which allowed for a shift in slope starting at minute 19, the time when the confederate spoke to endorse (confirming) or disavow (disconfirming) the beverage's effectiveness.

All models primarily sought to test differences by condition. We coded condition using both the dummy variable strategy, with the no confederate condition as the reference category, and the orthogonal strategy, described in the cross-sectional analyses section (these are equivalent but yield different comparisons). Other baseline covariates were included at Level 2 solely to reduce standard errors associated with condition-related coefficients and thereby to increase precision. In most cases, we report coefficients obtained from the regression output. To test specific hypotheses about intercepts and slopes when these were not directly available from the regression output, we used general linear hypothesis tests (denoted by chi-square tests) within the full longitudinal model.

Each final longitudinal model contained four random effects, one at Level 1 (representing residual variance across all timepoints, for each participant) and three at Level 2, to account for residual variance at each level, using an unstructured covariance structure that allowed the random effects for the intercept and primary slope (e.g., the slope that began at the timepoint at which time was mean-centered for the model) to covary. The Level-2 variance components consisted of a random intercept (representing residual variance in initial outcomes across participants), a random slope (representing residual variance in linear rate of change across participants), and their covariance (the covariance between the initial value of the outcome and slope across all participants).

Systolic Blood Pressure [T14-T24, Mean-centered at T14] Model

For systolic blood pressure (SBP), the key epoch of interest was from minute 19-24, the period of shared (confirming confederate condition) or unshared (disconfirming confederate condition) reality about the product. We analyzed effects during this post-shared reality period within a larger timeframe, to enable us to include the consumption and post-consumption time periods directly before the focal period of interest. Thus, for this analysis, we assessed changes in blood pressure from minute 14 to 24. We also controlled for average blood pressure from minute 1 to 13 at Level 2 (grand-mean centered within the full sample).

S1 Table presents the multilevel linear regression model for change in systolic blood pressure over the course of 10 minutes during the experiment, from minute 14 to minute 24. The best fitting model contained a shift in slope starting at minute 19, and also contained controls for blood pressure from minute 1 to minute 13 at Level 2. The parameters given in the S1 Table correspond to the following models, using the orthogonal coding strategy:

Level-1 Model:

$$SBP_{ij} = \pi_{0i} + \pi_{1i}MIN_{ij} + \pi_{2i}MIN\_CS\_S_{ij} + \varepsilon_{ij}$$

Level-2 Models (Orthogonal Coding):

$$\pi_{0i} = \gamma_{00} + \gamma_{01}COvsNC_i + \gamma_{02}IvsC_i + \gamma_{03}SBP\_T1T13_i + \delta_{0i}$$
$$\pi_{1i} = \gamma_{10} + \gamma_{11}COvsNC_i + \gamma_{12}IvsC_i + \gamma_{13}SBP\_T1T13_i + \delta_{1i}$$
$$\pi_{2i} = \gamma_{20} + \gamma_{21}COvsNC_i + \gamma_{22}IvsC_i + \gamma_{23}SBP\_T1T13_i$$

The outcome, SBP_{ij}, is the systolic blood pressure for participant *i* at minute *j* of the experiment. The Level-1 (within-subjects) predictor MIN_{ij} is the time in minutes when a measurement of systolic blood pressure (SBP) was taken, and represents the change in SBP over time (the slope, or average change for every one-minute increment). MIN_{ij} is centered on minute 14 of the experimental procedure, the time when participants begin drinking the AquaCharge beverage; thus the values of MIN_{ij} range from 0 (minute 14) to 10 (minute 24). $MIN\_CS\_S_{ij}$ represents a shift in slope starting at minute 19, the time when the confederate speaks in the confirming and disconfirming confederate conditions. It is 0 from minute 14 to 19, and increments by 1 thereafter, the same increment as the regular linear slope parameter MIN_{ij}.

Time	MIN	MIN_CS_S
14	0	0
15	1	0
16	2	0
17	3	0
18	4	0
19	5	0
20	6	1
21	7	2

22	8	3
23	9	4
24	10	5

At Level-2 (between-subjects), the two orthogonal condition variables are defined as in the cross-sectional analyses. SBP_T1T13_i is the average systolic blood pressure from minute 1 to minute 13 for participant *i*, grand-mean centered. We compute the effect of condition when this baseline blood pressure is set to 0 (due to mean centering, this corresponds to the average blood pressure for the sample during the T1 to T13 period).

The key coefficients of interest are γ_{12} and γ_{22}. The first is the difference in slope (change in SBP for every 1-minute increment) between the disconfirming and confirming confederate conditions during the period from minute 14 to minute 19, pre-confederate reaction to the product. The second is a difference in difference. It quantifies the condition difference in the shift in slope from the pre to post confederate reaction periods: it corresponds to the difference between the T24-T19 and T19-T14 slopes for the disconfirming confederate condition minus the difference between the T24-T19 and T19-T14 slopes for the confirming confederate condition. This model can be used to derive the difference in slope between the disconfirming and confirming confederate conditions from minute 19 to 24, which corresponds to:

$$MIN_{ij} \times IvsC_{ij} + MIN\_CS\_S_{ij} \times IvsC_{ij}$$

This is equivalent to $\gamma_{12} + \gamma_{22}$. By setting this equation to 0 and testing the null hypothesis that this difference is equal to 0 within the full longitudinal model (a post-hoc general linear hypothesis test), it is possible to test within the T14-mean-centered model whether the slopes for the disconfirming and confirming confederate conditions from T19 to T24 differ, the key question of interest.

For the dummy coding condition strategy, the same Level-1 model was used but different condition variables were used at Level 2:

Level-2 Models (Dummy Coding):

$$\pi_{0i} = \gamma_{00} + \gamma_{01}CvsCON_i + \gamma_{02}IvsCON_i + \gamma_{03}SBP\_T1T13_i + \delta_{0i}$$
$$\pi_{1i} = \gamma_{10} + \gamma_{11}CvsCON_i + \gamma_{12}IvsCON_i + \gamma_{13}SBP\_T1T13_i + \delta_{1i}$$
$$\pi_{2i} = \gamma_{20} + \gamma_{21}CvsCON_i + \gamma_{22}IvsCON_i + \gamma_{23}SBP\_T1T13_i$$

The key coefficients of interest are γ_{12} and γ_{12} and γ_{21} and γ_{22} but they now indicate different group comparisons than in the orthogonally-coded Level 2 model. γ_{12} and γ_{12} are the difference in slope (change in SBP for every 1-minute increment) between the confirming confederate and control (no confederate) conditions, and between the disconfirming confederate and control conditions, respectively, during the period from minute 14 to minute 19, pre-confederate reaction to the product. γ_{21} and γ_{22} are a difference in difference. They quantify the condition difference in the shift in slope from the pre to post confederate reaction periods, as a

function of condition: γ_{21} corresponds to the difference between the T24-T19 and T19-T14 slopes for the confirming confederate condition minus the difference between the T24-T19 and T19-T14 slopes for the no confederate condition. γ_{22} corresponds to the difference between the T24-T19 and T19-T14 slopes for the disconfirming confederate condition minus the difference between the T24-T19 and T19-T14 slopes for the no confederate condition. This model can be used to derive the difference in slope between the confirming confederate and no confederate conditions from minute 19 to 24 (equivalent to $\gamma_{11} + \gamma_{21}$):

$$MIN_{ij} \times CvsCON_{ij} + MIN\_CS\_S_{ij} \times CvsCON_{ij}$$

And the difference in slope between the disconfirming confederate and no confederate conditions from minute 19 to 24 (equivalent to $\gamma_{12} + \gamma_{22}$):

$$MIN_{ij} \times IvsCON_{ij} + MIN\_CS\_S_{ij} \times IvsCON_{ij}$$

By setting each equation to 0 and testing the null hypothesis (separately) that this difference is equal to 0 within the full T14 mean-centered longitudinal model (a post-hoc general linear hypothesis test), it is possible to test whether the slopes for each of the social influence conditions differ from the slope for the control (no confederate) condition from T19 to T24.

Systolic Blood Pressure [T14-T24, Mean-centered at T19]

As a check, we mean-centered time at minute 19, rather than minute 14, so that the slopes for T19-T24 could be obtained directly from the regression output (see S2 Table). In this model, the shift in slope occurred from T14 to T19. The time variables were thus structured as follows, where $MIN\_PW\_S2_{ij}$ represents the shift in slope during the post water consumption period (but prior to the confederate reaction period), and MIN_{ij} is now defined as the change in SBP from T19 to T24:

Time	MIN	MIN_PW_S2
14	-5	-5
15	-4	-4
16	-3	-3
17	-2	-2
18	-1	-1
19	0	0
20	1	0
21	2	0
22	3	0
23	4	0
24	5	0

This yielded the same results as the original model that was mean-centered at T14 (see S1 Table) but allowed us to obtain the slopes for the key time period, T19 to T24, directly from the regression model.

We report the longitudinal systolic blood pressure results for both models in the main text.

Subjective Alertness Longitudinal Model

S5 Table presents the multilevel linear regression model for change in subjective alertness over the course of the experiment, from minute 13 to minute 29. The best fitting model did not contain discontinuities in elevation or slope, as with SBP. However, it did contain controls for BMI and an index of caffeine expectancy at Level 2. The parameters given in the S5 Table correspond to the following models:

Level-1 Model:

$$MR\_ALERT_{ij} = \pi_{0i} + \pi_{1i} MIN_{ij} + \varepsilon_{ij}$$

Level-2 Models (Dummy Coding):

$$\pi_{0i} = \gamma_{00} + \gamma_{01} CvsCON_i + \gamma_{02} IvsCON_i + \gamma_{03} BMI_i + \gamma_{04} CAFF\_Exp_i + \delta_{0i}$$
$$\pi_{1i} = \gamma_{10} + \gamma_{11} CvsCON_i + \gamma_{12} IvsCON_i + \gamma_{13} BMI_i + \gamma_{14} CAFF\_Exp_i + \delta_{1i}$$

The outcome, MR_ALERT_{ij}, is the level of subjective alertness reported by participant *i* at minute *j* of the experiment, based on measurements taken at three timepoints (T13, T24, and T29). The Level-1 (within-subjects) predictor MIN_{ij} is the time in minutes when a measurement of subjective alertness was taken, and represents the change in subjective alertness over time (the slope, or average change for every one-minute increment). MIN_{ij} is centered on minute 13 of the experimental procedure; thus the values of MIN_{ij} range from 0 (minute 13) to 11 (minute 24) to 16 (minute 29).

At Level-2 (between-subjects), $CvsCON_i$ (=1 for the confirming confederate condition and 0 otherwise) and $IvsCON_i$ (=1 for the disconfirming confederate condition and 0 otherwise) are two dummy variables which represent the participant's experimental condition. BMI_i and $CAFF\_Exp_i$ are the subject's baseline body mass index and self-reported expectations of caffeine's effect on them, respectively. We compute the effect of condition when these covariates are set to 0 (due to mean centering, this corresponds to the average for the sample in each case).

The key coefficients of interest are γ_{11} and γ_{12}. The first is the difference in slope (change in alertness for every 1-minute increment) between the confirming confederate and no confederate conditions. The second is the difference in slope between the disconfirming confederate and no confederate conditions. To more easily compare the confirming and disconfirming confederate conditions, we used the orthogonal coding strategy for condition at Level 2:

Level-2 Models (Orthogonal Coding):

$$\pi_{0i} = \gamma_{00} + \gamma_{01} COvsNC_i + \gamma_{02} IvsC_i + \gamma_{03} BMI_i + \gamma_{04} CAFF\_Exp_i + \delta_{0i}$$
$$\pi_{1i} = \gamma_{10} + \gamma_{11} COvsNC_i + \gamma_{12} IvsC_i + \gamma_{13} BMI_i + \gamma_{14} CAFF\_Exp_i + \delta_{1i}$$

Here, the first condition variable (+0.5 for the confirming confederate and disconfirming confederate conditions; -1 for the no confederate condition) represents the difference in subjective alertness between subjects in the two social influence conditions and subjects in the no confederate condition. The second condition variable represents the difference in subjective alertness between the disconfirming (=+0.5) and confirming (=-0.5) confederate conditions. The key parameter is γ_{12}, the difference in slope between these two conditions. In the results below, we focus on the comparison between the disconfirming and confirming confederate conditions.

Subjective Alertness Longitudinal Results

S1 Fig displays the results of the subjective alertness longitudinal analyses. Below we report results with and without controls for baseline BMI and caffeine expectancy.

Basic Condition Only Model. At time 13, as expected, there was no difference in subjective alertness between subjects in the confirming (M_{adj}=3.33) and disconfirming (M_{adj}=3.32) conditions, *B*=--0.005, *z*=-0.03, *p*=0.977. Each condition exhibited evidence of a placebo effect, in the form of a significant positive slope. In the confirming confederate condition, subjective alertness increased by 0.57 scale points from minute 13 to minute 29 of the experiment (slope = 0.036/min), *B*=0.036, *z*=5.98, *P*<0.001. In the disconfirming confederate condition, subjective alertness still increased significantly over time, *B*=0.015, *z*=2.53, *P*=0.012, but only increased by 0.24 points during the same time period (slope=0.015/min). This difference in slopes was significant, *B*=-0.021, *z*=-2.43, *p*=0.015. By minute 29, subjective alertness was 3.90 for confirming participants and 3.56 for disconfirming participants, also a significant difference, $\chi(1)$=5.39, *p*=0.0203. In the no confederate condition, subjective alertness increased by 0.44 scale points from minute 13 to minute 19 (slope = 0.027/min), *B*=0.027, *z*=4.81, *P*<0.001, an intermediate level. However, this slope was not statistically different from either the confirming confederate condition, *B*=0.008, *z*=1, *p*=0.317, or the disconfirming confederate condition, *B*=-0.012, *z*=-1.48, *p*=0.138.

Final Model. When BMI and especially caffeine expectancy were controlled, the condition effects were slightly weaker and became marginally significant. At time 13, as expected, there was no difference in subjective alertness between subjects in the confirming (M_{adj}=3.33) and disconfirming (M_{adj}=3.30) conditions, *B*=-0.032, *z*=-0.19, *p*=0.848. In the confirming confederate condition, subjective alertness increased by 0.53 scale points from minute 13 to minute 29 of the experiment (slope = 0.033/min), *B*=0.033, *z*=6.33, *P*<0.001. In the disconfirming confederate condition, subjective alertness also significantly increased, *B*=0.018, *z*=3.22, *P*=0.001, but only by 0.29 points (slope=0.018/min). This difference in slopes was marginally significant, *B*=-0.015, *z*=-1.92, *p*=0.055. By minute 29, subjective alertness was 3.87 for confirming-condition participants and 3.60 for disconfirming-condition participants, also a marginally significant difference, $\chi(1)$=3.63, *p*=0.0568. The slope for the no confederate condition was again at an intermediate level, an increase of 0.36 over the entire 16-minute period (slope = 0.022/min), *B*=0.022, *z*=4.32, *P*<0.001. The difference in slopes between each of the two social influence conditions and the no confederate condition continued to be non-significant, *z*s<1.55, *p*s>0.13.
